# Comparative analyses of simple sequence repeats (SSRs) in 23 mosquito species genomes: Identification, characterization and distribution (Diptera: Culicidae)

**DOI:** 10.1111/1744-7917.12577

**Published:** 2018-04-06

**Authors:** Xiao‐Ting Wang, Yu‐Juan Zhang, Liang Qiao, Bin Chen

**Affiliations:** ^1^ Chongqing Key Laboratory of Vector Insects; Chongqing Key Laboratory of Animal Biology; Institute of Entomology and Molecular Biology Chongqing Normal University Chongqing China

**Keywords:** characterization, comparative analyses, distribution, mosquito, simple sequence repeats (SSRs), whole‐genome identification

## Abstract

Simple sequence repeats (SSRs) exist in both eukaryotic and prokaryotic genomes and are the most popular genetic markers, but the SSRs of mosquito genomes are still not well understood. In this study, we identified and analyzed the SSRs in 23 mosquito species using *Drosophila melanogaster* as reference at the whole‐genome level. The results show that SSR numbers (33 076–560 175/genome) and genome sizes (574.57–1342.21 Mb) are significantly positively correlated (*R* ^2^ = 0.8992, *P* < 0.01), but the correlation in individual species varies in these mosquito species. In six types of SSR, mono‐ to trinucleotide SSRs are dominant with cumulative percentages of 95.14%–99.00% and densities of 195.65/Mb–787.51/Mb, whereas tetra‐ to hexanucleotide SSRs are rare with 1.12%–4.22% and 3.76/Mb–40.23/Mb. The (A/T)n, (AC/GT)n and (AGC/GCT)n are the most frequent motifs in mononucleotide, dinucleotide and trinucleotide SSRs, respectively, and the motif frequencies of tetra‐ to hexanucleotide SSRs appear to be species‐specific. The 10–20 bp length of SSRs are dominant with the number of 110 561 ± 93 482 and the frequency of 87.25% ± 5.73% on average, and the number and frequency decline with the increase of length. Most SSRs (83.34% ± 7.72%) are located in intergenic regions, followed by intron regions (11.59% ± 5.59%), exon regions (3.74% ± 1.95%), and untranslated regions (1.32% ± 1.39%). The mono‐, di‐ and trinucleotide SSRs are the main SSRs in both gene regions (98.55% ± 0.85%) and exon regions (99.27% ± 0.52%). An average of 42.52% of total genes contains SSRs, and the preference for SSR occurrence in different gene subcategories are species‐specific. The study provides useful insights into the SSR diversity, characteristics and distribution in 23 mosquito species of genomes.

## Introduction

Simple sequence repeats (SSRs), also known as microsatellites, are 1–6 bp of tandem repeat nucleotides, and they exist in both protein coding regions and non‐coding regions in eukaryotic and prokaryotic genomes. Due to the high mutability of SSRs, which stem from their susceptibility to slippage events in DNA replication, SSRs provide an evolutionary mechanism for faster adaptation in response to environmental stress (Jiang *et al*., 2014; Willems *et al*., 2014). In comparison to other molecular markers, SSRs have many advantages, for example their high variability, co‐dominant mode of inheritance, multiple alleles and wide genome distribution (Schlötterer, 2004). These advantages make SSRs widely applied in genetic linkage mapping (Miao *et al*., 2005; Zhao *et al*., 2008), quantitative trait loci mapping (Shen *et al*., 2005), population genetics (Kim *et al*., 2008), genetic diversity analysis (Lehmann *et al*., 1996; Field *et al*., 1999; Manni *et al*., 2015) and comparative genomics (Behura & Severson, 2012, 2015). Early identification of SSRs mainly relied on the construction and screening of SSR‐enriched libraries (Zane *et al*., 2002), which were time‐consuming and only partially effective. In recent years, with the increasing number of genomes sequenced, *in silico* mining of SSR sequences from genome sequence databases has been widely used for SSR detection (Majumdar & Chatterjee, 2010), which is much more effective and comprehensive. The *in silico* mining of SSRs also allows for practical analysis of SSR distribution, putative function and evolution (Li *et al*., 2002). Whole‐genome SSR detection has been reported in plants such as *Oryza minuta* and *Or. punctata* (Wang *et al*., 2014), *Sesamum indicum* (Wei *et al*., 2014), and *Ziziphus jujuba* (Xiao *et al*., 2015) and in 30 marine animals (Jiang *et al*., 2014), six species of birds (Huang *et al*., 2016), six species of bovids (Qi *et al*., 2015), *Apis cerana* (Liu *et al*., 2016) and *Tribolium castaneum* (Demuth *et al*., 2007). The SSRs in coding sequences (CDs) of 25 insect species have been identified and comparatively analyzed (Behura & Severson, 2012).

Most mosquitoes are common vectors of infectious diseases, and threaten the health of human beings. Some SSR markers have been isolated and characterized using traditional techniques in *Anopheles minimus* (Bonizzoni *et al*., 2011), *An. sinensis* (Ma & Fan, 2008; Bonizzoni *et al*., 2011), *An. dirus* (Bonizzoni *et al*., 2011), *An. maculatus* (Rongnoparut *et al*., 1996) and *Aedes albopictus* (Porretta *et al*., 2006). The SSRs of *An. gambiae* have been identified at the whole‐genome level (Yu *et al*., 2005). As of January 2017, there have been 21 126 SSR sequences of insects reported in the National Center for Biotechnology Information (NCBI) database (http://www.ncbi.nlm.nih.gov/), of which 1966 SSRs belong to mosquitoes. Some SSR markers have been successfully applied to population studies in *Ae. aegypti* (Lovin *et al*., 2009), *An. sinensis* (Ma *et al*., 2011), *Ae. albopictus* (Manni *et al*., 2015) and *An. gambiae* (Lehmann *et al*., 1996; Field *et al*., 1999). The genomes of *An. gambiae* (Holt *et al*., 2002), *Culex quinquefasciatus* (Arensburger *et al*., 2010), *Ae. aegypti* (Nene *et al*., 2007) and *Ae. albopictus* (Chen *et al*., 2015) have been sequenced and annotated, and more recently 16 *Anopheles* genomes have been reported (Neafsey *et al*., 2015). A number of transcriptomes and mitochondrial genomes have also been sequenced and comparatively analyzed (e.g., Chen *et al*., 2014; Hua *et al*., 2016; Hao *et al*., 2017; Fang *et al*., 2018)

With the increasing of mosquito omics data, the diversity, characterization and functional analyses of a given group of genes are progressively increasing (He *et al*., 2016; Liu *et al*., 2018; Mei *et al*., 2018; Wang *et al*., 2018; Wu *et al*., 2018; Yan *et al*., 2018). However, the *in silico* SSR identification and analysis are relatively insufficient, and therefore the SSR features and the association with different species of mosquito genomes are still not well understood. Are SSR numbers and genome sizes corrected in different species? How do the frequency and density of different types of SSRs occur? Are they various among different species or taxa? What motifs are most frequent in different types of SSRs? What lengths of SSRs are dominant? Do the SSRs have any distribution differences among different regions of genomes? What proportion of genes contains SSRs? Are there any preferences for SSR occurrence in different functional categories of genes? To answer these questions, it is necessary to test different mosquito species with known phylogeny.

In this study, we comparatively analyzed the SSRs of 23 mosquito species at the whole‐genome level. This work involved SSR identification and classification; analyses of density, abundance, length, GC content and genomic distribution of SSRs; and the Gene Ontology (GO) enrichment of SSR‐containing genes and of all genes. The SSRs identified in the present study provide potentially important molecular markers for the study of population genetics, genetic mapping and regulatory mechanisms of functional genes in mosquito species. More importantly, this work provides useful insights into structure and distribution characteristics of SSRs as well as their variation patterns among different species.

## Materials and methods

### Genome sequences source

In this study, the genome sequences of 23 mosquito species in FASTA format and their annotation information in basefeatures format were downloaded from VectorBase (https://www.vectorbase.org/), and the genome sequences and annotation of *Drosophila melanogaster* (as reference) were from NCBI (http://www.ncbi.nlm.nih.gov/). All these genome sequences were assembled into scaffolds, except for *An. nili* which was assembled into contigs (Table 1). Among the 23 mosquito species, three belong to the family Culicinae, and the remaining 20 belong to *Anopheles* in the subfamily Anophelinae.

**Table 1:** Information on classification, genome database and genome annotation of 23 mosquito species and *Drosophila melanogaster* investigated in this study

		Genome source†		Genome annotation‡	
Family/subfamily genus/subgenus	Species	Version	Size (Mb)	Version	Size (Mb)
Drosophilidae	*D. melanogaster*	ISO1_MT	138.91	ISO1_MT	110.90
Culicidae/Culicinae					
*Aedes*	*Ae. aegypti*	AaegL3	1342.21	AaegL3.3	24.81
	*Ae. albopictus*	AaloF1	1868.07	AaloF1.1	38.17
*Culex*	*Cx. quinquefasciatus*	CpipJ2	574.57	CpipJ2.2	20.77
Culicidae/Anophelinae					
*Anopheles/Nyssorhynchus*	*An. darlingi*	AdarC3	132.94	AdarC3.2	11.00
	*An. albimanus*	AalbS1	165.33	AalbS1.2	11.45
*Anopheles/Anopheles*	*An. sinensis*	AsinC2	214.86	AsinC2.1	17.41
	*An. atroparvus*	AatrE1	217.57	AatrE1.2	13.36
*Anopheles/Cellia*	*An. farauti*	AfarF1	175.52	AfarF1.2	12.80
	*An. dirus A*	AdirW1	209.79	AdirW1.2	11.99
	*An. funestus*	AfunF1	218.45	AfunF1.2	12.20
	*An. minimus A*	AminM1	195.70	AminM1.2	12.02
	*An. culicifacies A*	AculA1	198.03	AculA1.2	13.88
	*An. maculatus*	AmacM1	141.20	AmacM1.2	17.10
	*An. stephensi*	AsteI2	216.26	AsteI2.2	15.12
	*An. epiroticus*	AepiE1	216.83	AepiE1.2	10.73
	*An. christyi*	AchrA1	169.04	AchrA1.2	13.46
	*An. melas*	AmelC1	222.01	AmelC1.2	15.24
	*An. merus*	AmerM1	244.34	AmerM1.2	13.06
	*An. quadriannulatus*	AquaS1	275.35	AquaS1.2	12.18
	*An. arabiensis*	AaraD1	239.13	AaraD1.2	12.94
	*An. gambiae*	AgamP4	268.44	AgamP4.2	20.36
	*An. coluzzii*	AcolM1	218.22	AcolM1.1	15.71
	*An. nili*	AnilD1	98.58		

^†^All genomes were assembled into scaffolds except for *An. nili* which was into contigs. All data were downloaded from VectorBase (https://www.vectorbase.org/downloads), except *D. melanogaster*, which was from National Center for Biotechnology information (NCBI) (https://www.ncbi.nlm.nih.gov/genome/?term = Dr.+melanogaster+).

^‡^All annotation files of genomes were in Basefeatures format, and all data were downloaded from Vector Base except for *D. melanogaster* from NCBI.

### Genome‐wide identification of SSRs

MISA (MIcroSAtellite, https://pgrc.ipk-gatersleben.de/misa/), was used to identify the SSRs in the 23 mosquito genomes and *D. melanogaster* investigated in this study. The software has high site detection with accuracy rates, and with export results including the numbers of different types of SSRs and the position and length (motif bp × number of motif) of each (Thiel *et al*., 2003). The minimum number of repeats was set as 10 for mononucleotide SSR detection in the running of the software, six for dinucleotide SSRs, and five for tri‐, tetra‐, penta‐ and hexa‐nucleotide SSRs (Zhao *et al*., 2015). In the density calculation of different SSR motifs, different sequences produced from shifted permutations and/or reverse complements were treated as a single motif. For example, AAC, ACA, CAA, TTG, TGT and GTT were treated as a single motif AAC because these repeats were shifted permutations and/or reverse complements (Jurka & Pethiyagoda, 1995; Li *et al*., 2009). The shortest basic sequence was treated as the motif of the SSR with any number of repeats of the basic sequence. For example, the motif (AGAG)_9_ is AG, and thus the (AGAG)_9_ was treated as (AG)_18_.

### Characteristics of SSRs in mosquitoes

The density (SSR quantity per Mb of genomic sequence) and abundance (proportion of a given type of SSR among total SSRs) were used to measure the frequency of SSR in genomes (Wang *et al*., 2015b). The length of SSRs was divided into 10 groups with 10 bp intervals, namely 10–20, 21–30, 31–40, 41–50, 51–60, 61–70, 71–80, 81–90, 91–100 and 101+ bp (Zhao *et al*., 2015). Excel 2010 was used to count the number of each length group of SSR and to calculate the relationship between the length and number of SSRs. A Perl script was specially written to calculate the GC‐content of each SSR sequence and the average of GC‐content of all types of SSR.

### SSR distribution through different genomic regions

SSRs are ubiquitous, but not evenly distributed in inter‐genic, intron, exon and untranslated (UTR) regions of sequence (Kashi & King, 2006). Different species may have different motif frequency distributions, and SSRs in different genomic regions may have different features and thus perform varied functions (Levinson & Gutman, 1987; Schlötterer & Tautz, 1992; Sonah *et al*., 2011). In the present study, an additional Perl script was also written to recognize SSRs in inter‐genic, intron, exon and UTR regions based on the SSR position information obtained and genome annotation information downloaded, and a count of the SSR numbers in each region.

### GO enrichment of SSR‐containing genes and all genes

Based on the identification of the SSRs in different genomic regions, we sorted out SSR‐containing genes from the annotation documents. We then extracted the GO annotation numbers of SSR‐containing genes and of all genes from VectorBase for each species. Using WEGO (http://wego.genomics.org.cn/cgi-bin/wego/index.pl) and the GO annotation numbers obtained, we created a functional classification of SSR‐containing genes and analyzed the number and proportion of each functional category of SSR‐containing genes in comparison to all annotated genes for each species.

## Results and discussion

### SSR numbers in 23 mosquito species genomes

**Figure 1:**
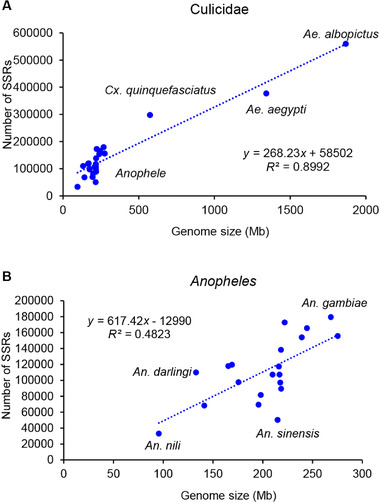
Relationship of genome size (Mb) and number of simple sequence repeats (SSRs) in 23 mosquito species. (A) A positive correlation was detected in the 23 mosquito species (*R* ^2^ = 0.8992, *P* < 0.01). (B) A positive correlation was detected in the 20 *Anopheles* mosquito species (*R* ^2^ = 0.4823, *P* < 0.05).

We identified the SSRs in the genomes of 23 mosquito species and *D. melanogaster*, and the information and genome position of each SSR are available for each species from the authors on request. The numbers of SSRs ranged from 33 076 for *An. nili*, which had the smallest genome size of 95.58 Mb, to 560 175 for *Ae. albopictus*, which had the largest genome size of 1 868.07 Mb. The SSR numbers and the genome sizes for the 23 mosquito species were significantly positively correlated (Fig. 1A, *R* ^2^ = 0.8992, *P* < 0.01). This phenomenon was the same as that for 30 marine species reported (Jiang *et al*., 2014) and six bovid species (Qi *et al*., 2015), in which the genome sizes were also correlated with the SSR numbers. SSRs are supposed to contribute to larger genome sizes since the accumulation of genes is not responsible for large differences in genome size (Jiang *et al*., 2014). The positive linear correlation indicated that SSR repetition in genomes in part reflects a species’ genome size (Hancock, 1996; Tóth *et al*., 2000; Katti *et al*., 2001).

The genome sizes for three species in Culicinae, *Ae. albopictus* (1868.07 Mb), *Ae. aegypti* (1342.21 Mb) and *Cx. quinquefasciatus* (574.57 Mb), with an average of 1261.62 ± 531.1 Mb (mean ± SD), were much bigger than those of the 20 *Anopheles* species in Anophelinae (201.88 ± 43.09 Mb), and correspondingly the SSR numbers of these three species (411 573 ± 109 989) were much larger than those of the 20 *Anopheles* species (111 637 ± 39 542) (Fig. 1A). The SSR numbers and the genome sizes for the three Culicinae species were highly positively correlated (*R* ^2^ = 0.9508, *P* < 0.01); they were significantly correlated for the 20 *Anopheles* species (Fig. 1B, *R* ^2^ = 0.4823, *P* < 0.05). For some *Anopheles* species, there were some exceptions for the correlation between the genome size and SSR number. For example, the genome size of *An. sinensis* (214.86 Mb) was bigger than that of nine *Anopheles* species, *An. nili* (98.58 Mb), *An. darlingi* (132.94 Mb), *An. maculatus* (141.20 Mb), *An. albimanus* (165.33 Mb), *An. farauti* (175.52 Mb), *An. christyi* (169.04 Mb), *An. mininus A* (195.70 Mb), *An. culicifacies A* (198.03 Mb) and *An. atroparvus* (217.57 Mb), whereas the SSR number (50 397) of *An. sinensis* was only larger than that of *An. nili* (33 076). These differences of genome size reflect the characteristics of the two subfamilies at the genomic level. The SSRs were thought to stem from the slippage events in DNA replication in response of faster adaptation in response to environmental stress (Jiang *et al*., 2014; Willems *et al*., 2014). The bigger genome size provides more chances for the birth of SSRs; therefore the overall correlations between the genome size and SSR number are reasonable. The exception of the correlation in some *Anopheles* species might reflect the difference of environment in which species live.

For mosquitoes, six SSRs were isolated in *Ae. albopictus* using an enriched genomic library technique (Porretta *et al*., 2006), and 20, 21 and 13 SSRs were identified in *An. minimus*, *An. sinensis* and *An. dirus*, respectively, using experimental techniques (Bonizzoni *et al*., 2011). There were 818, 5582 and 2976 simple sequence coding repeats detected in *Ae. aegypti*, *An. gambiae* and *Cx. quinquefasciatus* based on coding sequences, respectively (Behura & Severson, 2012). In *An. sinensis*, 252 SSRs were isolated using *Sau*LA sequence as primer and ligated DNA as template to construct a genomic library, and further investigation of 20 SSRs showed that 14 of them were cleanly amplified and polymorphic (Ma & Fan, 2008). Among 23 SSRs detected using probes in *An. maculatus*, four SSRs were selected to perform polymerase chain reaction (PCR) analysis, and they all showed a high level of polymorphism in a *An. maculatus* population (Rongnoparut *et al*., 1996). In the present study, 69 478, 50 397, 107 165, 179 406, 68 244, 560 175, 377 081 and 297 463 SSRs were identified and analyzed from *An. minimus*, *An. sinensis*, *An. dirus*, *An. gambiae*, *An. maculatus*, *Ae. albopictus*, *Ae. aegypti* and *Cx. quinquefasciatus* at the whole‐genome level (including non‐CDs), respectively (Fig. 1). In comparison, these SSR numbers detected are much larger than those earlier reported in corresponding species, and the characteristics of these SSRs are also explored in more detail. Although the bioinformatics method is highly effective and fast in the detection of SSR loci, these SSRs need to be confirmed by performing PCR and sequencing techniques in practical application due to possible inaccuracy from bioinformatics analysis and genetic polymorphism from different samples.

### Numbers and density of six types of SSRs in 23 mosquito genomes

**Figure 2:**
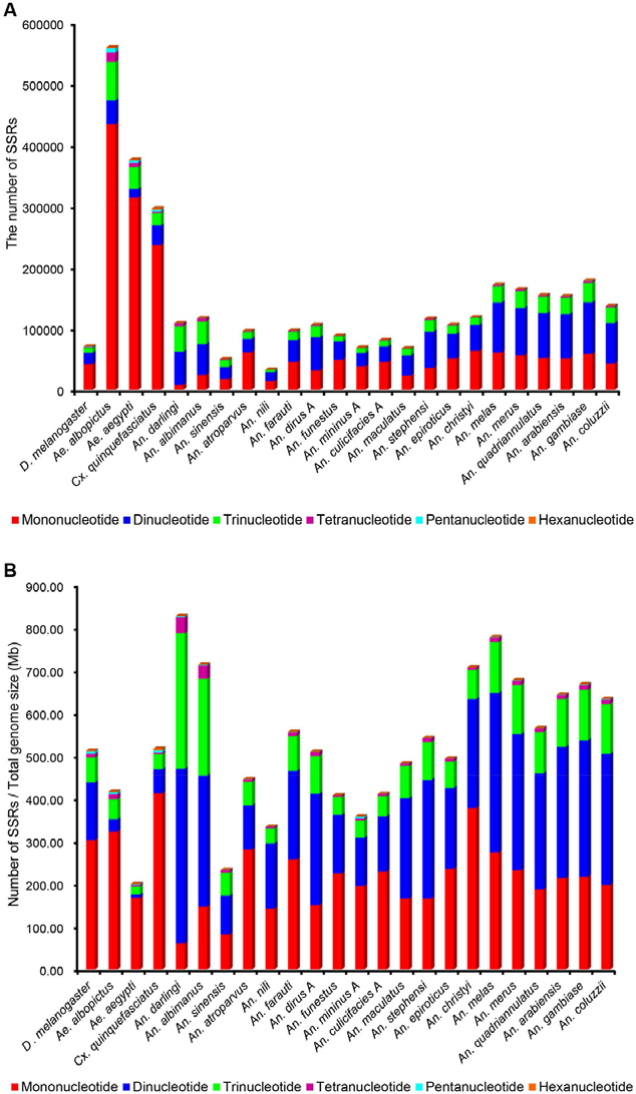
Abundance of mono‐ to hexanucleotide simple sequence repeats (SSRs) in genomes of 23 mosquito species and *Drosophila melanogaster*. (A) The *x*‐axis indicates the species of genome, and the *y*‐axis indicates the number of mono‐ to hexanucleotide SSRs. (B) The number of mono‐ to hexanucleotide SSRs per 1 Mb of genome sequence.

There were six types of SSR units, mono‐ to hexanucleotide, present in the 23 mosquito genomes, the same as reported in other genomes (Wei *et al*., 2007; Qi *et al*., 2015). In the six types of SSR, the cumulative numbers of mono‐ to trinucleotide SSRs occupied 95.14% (*An. darlingi*, 104 691 SSRs) to 99.00% (*An. funestus*, 88 461) of the total number of SSRs in the 23 mosquito species and in *D. melanogaster*, whereas the tetra‐ to hexanucleotide SSRs only made up 1.12% (*An. nili*, 371) to 4.22% (*Ae. albopictus*, 23 657) (Fig. 2A). These results suggested that the mono‐ to trinucleotide SSRs were the dominant types, and the tetra‐ to hexanucleotide SSRs were quite rare in these mosquito genomes. These results are consistent with earlier reports in six bovid species with mono‐ to trinucleotide SSRs comprising 82.37% of the total on average (Qi *et al*., 2015), and in the *Phyllostachys edulis*, *Zea mays*, *Or. sativa*, *Sorghum bicolor*, *Brachypodium distachyon* and *Arabidopsis thaliana* genomes with mono‐ to trinucleotide SSRs comprising 94.05%, 97.45%, 98.21%, 90.26%, 88.48% and 82.25% of the total, respectively (Zhao *et al*., 2015). Nevertheless, hexanucleotide SSRs are the dominant type in 30 marine animals (comprising 33.22%, Jiang *et al*., 2014), 10 Poaceae species (58.82%, Wang *et al*., 2015b) and three Gossypium species (39.4%, Wang *et al*., 2015a). The occurrence frequency of different types of SSRs seems characteristic of different individual species, which needs further taxonomic clarification given the increasing number of SSR investigations at the genome level.

The cumulative density of mono‐ to trinucleotide SSRs ranged from 195.65/Mb (*Ae. aegypti*) to 787.51/Mb (*An. darlingi*), while the density of tetra‐ to hexanucleotide SSRs was only 3.76/Mb (*An. nili*) to 40.23/Mb (*An. darlingi*) (Fig. 2B). For mononucleotide SSRs, the average density in the three species of the Culicinae (302.24/Mb) was about 1.5‐times larger than that of *Anopheles* species (203.31/Mb). For dinucleotide SSRs, the average density (30.82/Mb) was eight times smaller than that of the 20 *Anopheles* species (237.83/Mb). For trinucleotide SSRs, the average density (33.65/Mb) was three times smaller than that of *Anopheles* species (97.86/Mb). The average density for all six types of SSRs (378.97/Mb) was about 1.5 times smaller than that of *Anopheles* species (550.22/Mb). The results showed that the density of different types of SSRs at the genome level was also specific to the Culicinae and Anophelinae subfamilies.

### Motif frequency of SSRs in 23 mosquito species genomes

The occurrence frequency of motifs in SSRs varied in the 23 mosquito species and in *D. melanogaster* (Table S1). For mononucleotide SSRs, the motif (A/T)n was predominant with an average frequency of 84.83% ± 7.90%, ranging from 70.88% (*An. darlingi*) to 97.73% (*An. nili*), while the motif (C/G)n was far rarer with an average of 15.17% ± 7.90% (2.27% in *An. nili* to 29.12% in *An. darlingi*) in these genomes. This result is consistent with earlier reports for the rhesus monkey (Xu *et al*., 2016), six bovid species (Qi *et al*., 2015) and six bird species (Huang *et al*., 2016), in which (A/T)n was also the predominant motif in mononucleotide SSRs, comprising 99.55%, 93.27% and 87.7% of the total on average, respectively. The (A/T)n predominance in mononucleotide SSRs might stem from mutations or transpositions of poly‐A repeats (Tóth *et al*., 2000; Coenye & Vandamme, 2005). Nowadays, the mononucleotide SSRs are not used as molecular markers any more in the population studies due to instability in PCR amplification. In the dinucleotide SSRs, the motif (AC/GT)n was the most frequent (average of 62.35% ± 10.45%), followed by (AG/CT)n (29.94% ± 7.01%), (AT/AT)n (6.03% ± 9.64%), and (CG/CG)n (1.81% ± 1.04%), except for (AT/AT)n in *D. melanogaster* (31.41%) and *Ae. aegypti* (31.41%). The result is consistent with an earlier study in *An. gambiae*, in which the (AC/GT)n was a little more than two times as abundant, much as (AG/CT)n, and (AT/AT)n and (CG/CG)n were rare (Yu *et al*., 2005). Earlier works have shown that the frequency is variable in different groups of organisms, for example (AC/GT)n, (AG/CT)n and (AT/AT)n are predominant in 30 marine animals (average of 65.62%, Jiang *et al*., 2014), in six bamboo species (45.39%, Zhao *et al*., 2015) and in six bird species (46.92%, Huang *et al*., 2016), respectively, while (GC/GC)n is the lowest in all these groups. Due to high variation, dinucleotide SSRs are often used as molecular markers, for example the motif (AC)n has been used for population genetics studies in *An. maculatus* (Rongnoparut *et al*., 1996), *Ae. albopictus* (Manni *et al*., 2015) and *An. gambiae* (Field *et al*., 1999). The motif (AGC/GCT)n in trinucleotide SSRs was predominant (average of 33.01% ± 6.25%) in the present study. This is also consistent with earlier work in *An. gambiae* in which the (AGC/GCT)n predominated, while the (ACG/CGT)n, (ACT/AGT)n, (AGG/CCT)n and (CCG/CGG)n were rare. However, (AAG/CTT)n, (ACG/CGT)n and (AAT/ATT)n are predominant in six bamboo species (23.71%, Zhao *et al*., 2015), six bovid species (36.76%, Qi *et al*., 2015) and six bird species (30.97%, Huang *et al*., 2016), respectively. Also, the trinucleotide SSRs are often used as molecular markers, for example the motifs (CTG)n, (TCC)n and (CAC)n have been used as molecular markers in *An. sinensis* (Ma & Fan, 2008), the motif (AGC)n has been used in *An. gambiae* (Lehmann *et al*., 1996) and *Ae. aegypti* for population genetics studies (Lovin *et al*., 2009).

The motif frequencies of tetra‐ to hexanucleotide SSRs were not as conserved as those in mono‐ to trinucleotide SSRs, and appeared to be species‐specific in the present study. The motif (AAAT/ATTT)n was the most frequent tetranucleotide SSR in 18 mosquito species (average frequency 26.79% ± 10.83%), but not in *Cx. quinquefaciatus*, *An. darlingi*, *An. albimanus*, *An. nili*, *An. christyi* and *D. melanogaster*. Both (AAATG/CATTT)n and (AAAAC/GTTTT)n were the most frequent pentanucleotide SSRs in six mosquito species (average frequencies 26.98% ± 8.32%, 13.93% ± 3.34%), but there was no common motif in the remaining 12 species. For the hexanucleotide SSRs, the motif (AACAGC/GCTGTT)n was predominant in seven mosquito species (average 24.57% ± 5.62%), (AAGTAG/CTACTT)n was dominant in *An. mininus A* (18.25%), *An. maculatus* (23.08%) and *An. stephensi* (17.14%), and there was no common motif in the other species. The high variation of motif occurrence frequency in tetra‐ to hexanucleotide SSRs is consistent with earlier investigations, for example there are four main motifs, (AAAT/ATTT)n (27.14%), (AACTG/CAGTT)n (40.06%), (AGTTC/GAACT)n (39.49%) and (AAACAA/TTGTTT)n (16.35%) in six bovid species (Qi *et al*., 2015), and three main motifs, (AAAC/GTTT)n (33.0%), (AAACA/TGTTT)n (20.52%) and (AAAGAA/TTCTTT)n (14.42%) in six bird species (Huang *et al*., 2016).

### Length, density and GC content of the SSRs

**Figure 3:**
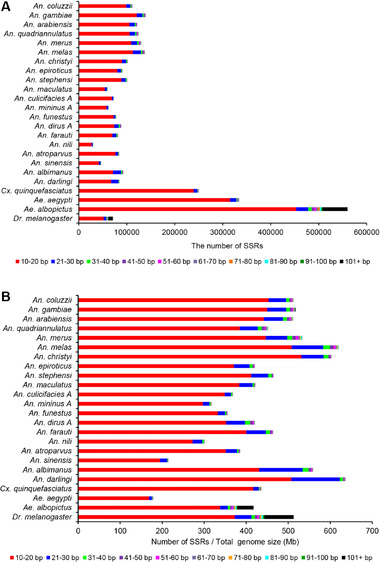
Length variation of simple sequence repeats (SSRs) in genomes of 23 mosquito species and *Drosophila melanogaster*. (A) The numbers of different lengths of SSRs in the 23 species investigated. (B) The number of different lengths of SSRs per 1 Mb of genome sequence.

The lengths of SSRs were divided into 10 different groups (10 bp intervals from 10–100 bp and 101+ bp) for the 23 mosquito species and *D. melanogaster* (Fig. 3). Generally, the number of SSRs declined with the increase of length across these groups. The 10–20 bp length was predominant with an average number of 110 561 ± 93 482 and an occurrence frequency of 87.25% ± 5.73% of the total number of SSRs in all 24 genomes (Fig. 3A). Following this group were 21–30 bp (average 9455 ± 5362, frequency 8.52% ± 3.70%), 31–40 bp (1786 ± 1393, 1.5% ± 0.68%) and 41–50 bp (984 ± 1050, 0.73% ± 0.46%), and the 101+ bp group of SSRs had the least average number at 96 ± 189 and an occurrence frequency of 0.06% ± 0.09% (Fig. 3A). This result is consistent with that in six bamboo species, in which 10–20 bp SSRs are also predominant (comprising an average of 85% of total SSRs) (Zhao *et al*., 2015). The variation of occurrence density (number of SSRs/total genome size in Mb) was largely consistent with the SSR number variation in these 24 species. The 10–20 bp SSRs had the highest density (average of 381.81 ± 87.49/Mb), followed by 21–30 bp (40.39 ± 26.08/Mb), 31–40 bp (7.11 ± 4.14/Mb) and 41–50 bp (3.58 ± 2.37/Mb), and the 101+ bp of SSRs had the lowest density (0.25 ± 0.43/Mb) (Fig. 3B). In earlier studies, SSRs with a length larger than 20 bp were defined as class I, which are characteristically low frequency and of high instability. Class I SSRs were thought to receive greater selection pressure and be more likely to be converted into shorter SSRs. Those SSRs with a length less than or equal to 20 bp were defined as class II, believed to be more stable and more suitable for molecular markers (Cho *et al*., 2000; Temnykh *et al*., 2004; Zhao *et al*., 2015).

The percent GC‐contents were calculated for mono‐ to hexanucleotide SSRs in the 20 *Anopheles* species, three Culicinae species, and for *D. melanogaster* (Table 2). The results showed that the average GC‐content values (6.77%–49.78%) in the three groups were each lower than those of AT‐content (50.22%–93.23%), except for the trinucleotide SSRs for the 20 *Anopheles* species in which the average GC‐content value (52.39%) was higher than the AT‐content (47.61%). In the six types of SSRs, the trinucleotide SSRs had the highest GC‐content values (52.39%, 44.02% and 45.82%), and the mononucleotide SSRs had the lowest GC‐content values (16.66%, 6.77%, and 8.49%) for *Anopheles*, Culicinae and *D. melanogaster* groups, respectively. Earlier whole‐genome studies of SSRs for other species have also shown that GC contents are lower than AT contents; for example, six bovid species (Qi *et al*., 2015) and rhesus monkey (Xu *et al*., 2016) have average GC contents of 27.39% and 16.52%, respectively. The methylation of CpG islands might produce mutations from cytosine (C) to thymine (T) by deamination (Schorderet & Gartler, 1992), and the rich AT‐content in SSRs may reduce the annealing temperature, which would increase the AT‐content after DNA replication slippage (Xu *et al*., 2016). The lower GC‐content has been reported to covary with genomic properties, such as DNA bendability (Vinogradov, 2001), the ability for B‐Z transition (Vinogradov, 2003), and replication regulation or expression timing (Hiratani *et al*., 2004). The relationship between GC‐content in SSRs and SSR polymorphisms might guide the choice of SSR markers (Glenn *et al*., 1996).

**Table 2:** Percent GC‐content of mono‐ to hexanucleotide simple sequence repeats in genomes of 23 mosquito species and *Drosophila melanogaster*

	Percent GC‐content					
Species	Mononucleotide	Dinucleotide	Trinucleotide	Tetranucleotide	Pentanucleotide	Hexanucleotide
*Anopheles darlingi*	29.08	50.96	59.35	62.94	57.15	57.90
*An. albimanus*	22.21	51.08	60.34	63.91	61.42	58.86
*An. sinensis*	13.71	49.52	53.89	40.04	28.89	46.77
*An. atroparvus*	12.97	49.24	56.48	41.14	34.29	53.00
*An. nili*	2.26	48.16	50.59	47.87	37.14	36.67
*An. farauti*	10.97	49.80	58.47	54.90	44.49	50.00
*An. dirus A*	19.87	51.49	56.63	42.91	30.85	38.98
*An. funestus*	7.49	49.44	46.27	30.37	22.03	36.46
*An. mininus A*	9.87	48.76	46.43	31.36	23.23	28.79
*An. culicifacies A*	6.17	49.21	46.90	31.75	21.33	33.33
*An. maculatus*	6.13	50.17	51.96	38.59	23.57	36.36
*An. stephensi*	19.49	49.95	51.82	41.75	36.73	40.32
*An. epiroticus*	21.31	49.62	52.27	37.49	28.97	44.36
*An. christyi*	10.17	48.41	48.74	39.16	30.70	40.07
*An. melas*	25.74	49.50	51.95	36.30	22.19	37.04
*An. merus*	24.93	50.04	51.29	37.32	21.75	37.59
*An. quadriannulatus*	23.34	50.23	51.82	36.42	21.87	32.07
*An. arabiensis*	23.24	50.15	51.49	35.74	23.54	32.58
*An. gambiae*	24.14	49.78	50.11	33.81	21.78	29.79
*An. coluzzii*	20.13	50.09	51.05	35.78	19.54	31.27
*Anopheles* average	16.66	49.78	52.39	40.98	30.57	40.11
*Aedes albopictus*	7.72	42.25	43.84	27.03	35.93	36.81
*Ae. aegypti*	4.38	30.80	35.09	11.94	33.41	41.77
*Culex quinquefasciatus*	8.22	48.25	53.13	28.14	25.48	34.86
Culicinae average	6.77	40.43	44.02	22.37	31.61	37.81
*D. melanogaster*	8.49	34.61	45.82	38.71	33.52	45.60

### Distribution of SSRs in different genomic regions

**Figure 4:**
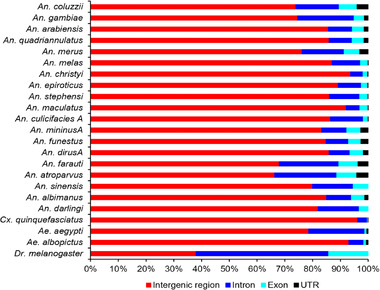
Percentage of simple sequence repeat (SSR) numbers in different genomic regions of 22 mosquito species and *Drosophila melanogaster*.

The distribution of SSRs varied in different genomic regions in the 22 mosquito species and in *D. melanogaster*. An average of 83.34% ± 7.72% SSRs were located in intergenic regions, followed by intron regions (average, 11.59% ± 5.59%), exon regions (3.74% ± 1.95%) and UTRs (1.32% ± 1.39%) (Fig. 4). An earlier study on *An. gambiae* genome showed that the SSRs in exons of all chromosomes were less abundant than in introns and intergenic regions except for mono‐ and dimer repeats in exons of chromosome 2L (Yu *et al*., 2005). The highest proportion of SSRs was also reported in the intergenic regions of six bamboo species (average 71.17%) (Zhao *et al*., 2015) and six bird species (84.93%) (Huang *et al*., 2016), whereas the majority of SSRs exist in gene regions in *D. melanogaster* (62.0%). This suggests that the distribution of SSRs in different genomic regions is specific for different species. SSRs in different regions show different functions. SSRs in intronic regions can affect gene regulation, messenger RNA splicing, and gene silencing (Li *et al*., 2004). SSRs in exonic regions can affect the activation of a gene and the truncation of a protein product, and SSRs in UTRs can affect gene transcription and regulation (Lawson & Zhang, 2006).

**Figure 5:**
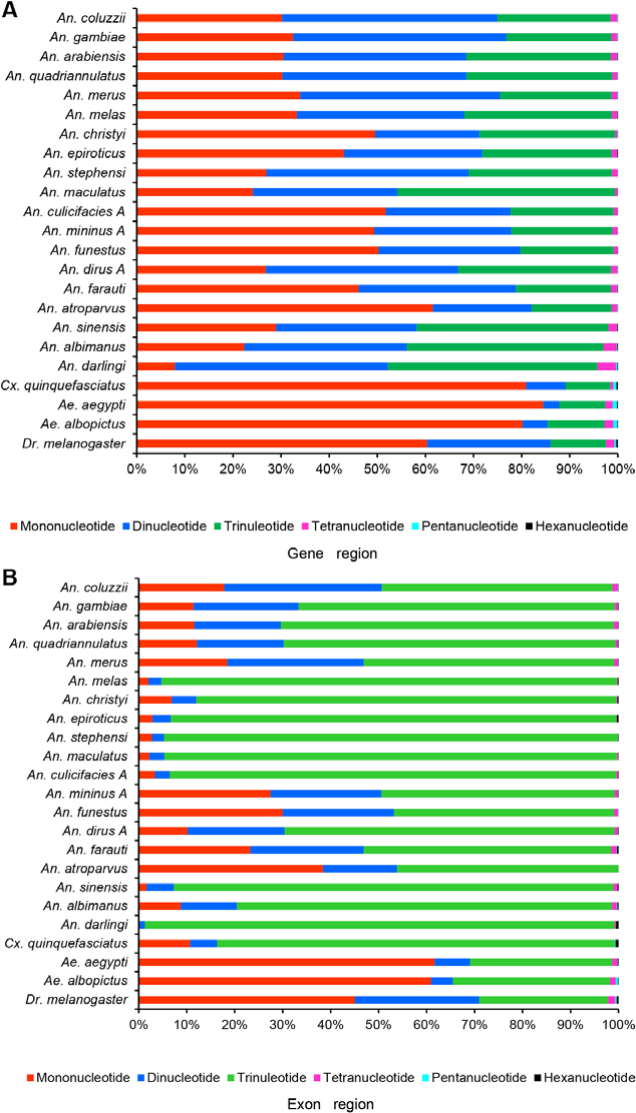
Percentage of mono‐ to hexanucleotide simple sequence repeats (SSRs) in 22 mosquito species and *Drosophila melanogaster*. (A) For gene regions (including intron, exon and untranslated regions). (B) For exon regions only.

Further analyses showed that the mono‐, di‐ and trinucleotide SSRs were the main components in both gene regions and exon regions in the species investigated, comprising an average of 98.55% ± 0.85% (Fig. 5A) and 99.27% ± 0.52% (Fig. 5B) of the total, respectively. The tetra‐ to hexanucleotide SSRs were relatively less, making up an average of 1.45% ± 0.85% in gene regions and 0.73% ± 0.52% in exon regions, respectively. The result is consistent with the identification of simple sequence coding repeats from CDs in *Ae. aegypti*, *An. gambiae* and *Cx. quinquefasciatus*, in which the mono‐, di‐ and trinucleotide SSRs make up 91.43%, 95.56% and 92.95%, respectively (Behura & Severson, 2012). Interestingly, the trinucleotide SSRs accounted for more than half of the total SSRs in exon regions in the 20 *Anopheles* species (average of 73.21% ± 23.05%) and in *Cx. quinquefasciatus* (82.86%), but less than half in *Ae. aegypti* (29.60%), *Ae. albopictus* (33.72%) and *D. melanogaster* (26.79%). Earlier whole‐genome SSR studies have also shown a trinucleotide SSR preference in exon regions in *Ap. cerana* (66.6% SSRs in exon region), *Ap. mellifera* (76.7%) (Liu *et al*., 2016) and *Laccaria bicolor* (41%) (Labbé *et al*., 2011). SSRs in exon regions may affect the evolution of protein structure and function (Majumdar & Chatterjee, 2010). The trinucleotide SSR preference in exon regions may inhibit other types of SSRs and thereby reduce the incidence of frameshift mutations in exons (Metzgar *et al*., 2000; Labbé *et al*., 2011).

### GO enrichments between SSR‐containing genes and all genes

For understanding the characteristics of SSR‐containing genes, and the differences of SSR‐containing genes in mosquito genomes, we compared functional annotations between SSR‐containing genes and all genes in 21 species with GO annotation by GO enrichment (*An. nili* and *An. stephensi* were not included due to a lack of GO numbers). As a result, an average of 42.52% of total genes contained SSRs in the 21 mosquito genomes. The number of SSR‐containing genes ranged from 2669 (14.07% of total genes) in *Cx*. *quinquefasciatus* to 8319 (57.14%) in *An*. *coluzzii*. In the three main GO categories (Cellular Component, Molecular Function and Biological Process) in the GO enrichment, the subcategories and percentage genes in most subcategories were highly similar for both SSR‐containing genes and all genes in the 21 mosquito species genomes (Fig. S1).

SSR occurrence showed obvious differences in several functional subcategories of genes. In the metallochaperone subcategory of the Molecular Function category, there were only four mosquito genomes (*Ae. albopictus*, *Ae. aegypti*, *Cx. quinquefasciatus*, *An. sinensis*) which contained SSRs, while the other 17 did not. In the protein tag subcategory of the Molecular Function category, there were 15 mosquito species that contained SSRs, and the remaining six (*An. darlingi*, *An. sinensis*, *An. dirus A*, *An. funestus*, *An. culicifacies A* and *An. maculatus*) did not. In the viral reproduction subcategory of the Biological Process category, three species of mosquito (*Ae. aegypti*, *An. albimanus*, *An. mininus A*) contained SSRs, whereas the remaining 18 did not. The reason that preference for SSR occurrence differed in these subcategories remains to be further studied with a wider range of species. An earlier comparative analysis of GO enrichment between genes containing the motif (ATGTAC/GTACAT)n and all genes in marine species showed that the genes containing the motif are involved in evolution (Jiang *et al*., 2014).

## Conclusion

The results of this study provide useful insights into the SSR diversity, characteristics and distribution in 23 mosquito species of genomes. The SSR repetition in genomes partially reflects a species’ genome size. The mono‐ to hexanucleotide SSRs are dominant, but the occurrence percentage and density of each type of SSR vary among different taxa. The SSRs with motif (AC/GT)n or (AGC/GCT)n with a length less than or equal to 20 bp would be better molecular markers. Most SSRs are distributed in intergenic regions, and the mono‐, di‐ and trinucleotide SSRs are the main SSRs in both gene regions and exon regions. This study lays an important basis for the better understanding of SSRs and the selection of SSR molecular markers in mosquitoes.

## Disclosure

The authors declare no conflict of interest.

## Supporting information


**Fig. S1**. GO classifications of SSR‐containing genes and all genes in 21 mosquito species.

**Table S1**. The most frequent SSR motifs in the 23 mosquito species and *D. melanogaster* genomes.
