# Supplementary material for: Comparative analyses of simple sequence repeats (SSRs) in 23 mosquito species genomes: Identification, characterization and distribution (Diptera: Culicidae)
Source: Insect Sci. 2018 Apr 6;26(4):607–19. doi: 10.1111/1744-7917.12577 (PMC7379697; doi:10.1111/1744-7917.12577)
Supplement: Supplementary file 1 — Fig. S1. GO classifications of SSR‐containing genes and all genes in 21 mosquito species. [file INS-26-607-s001.pdf]

Fig S1 GO classifications of SSR-containing genes and all genes in 21 mosquito species.

Ae. aegypti

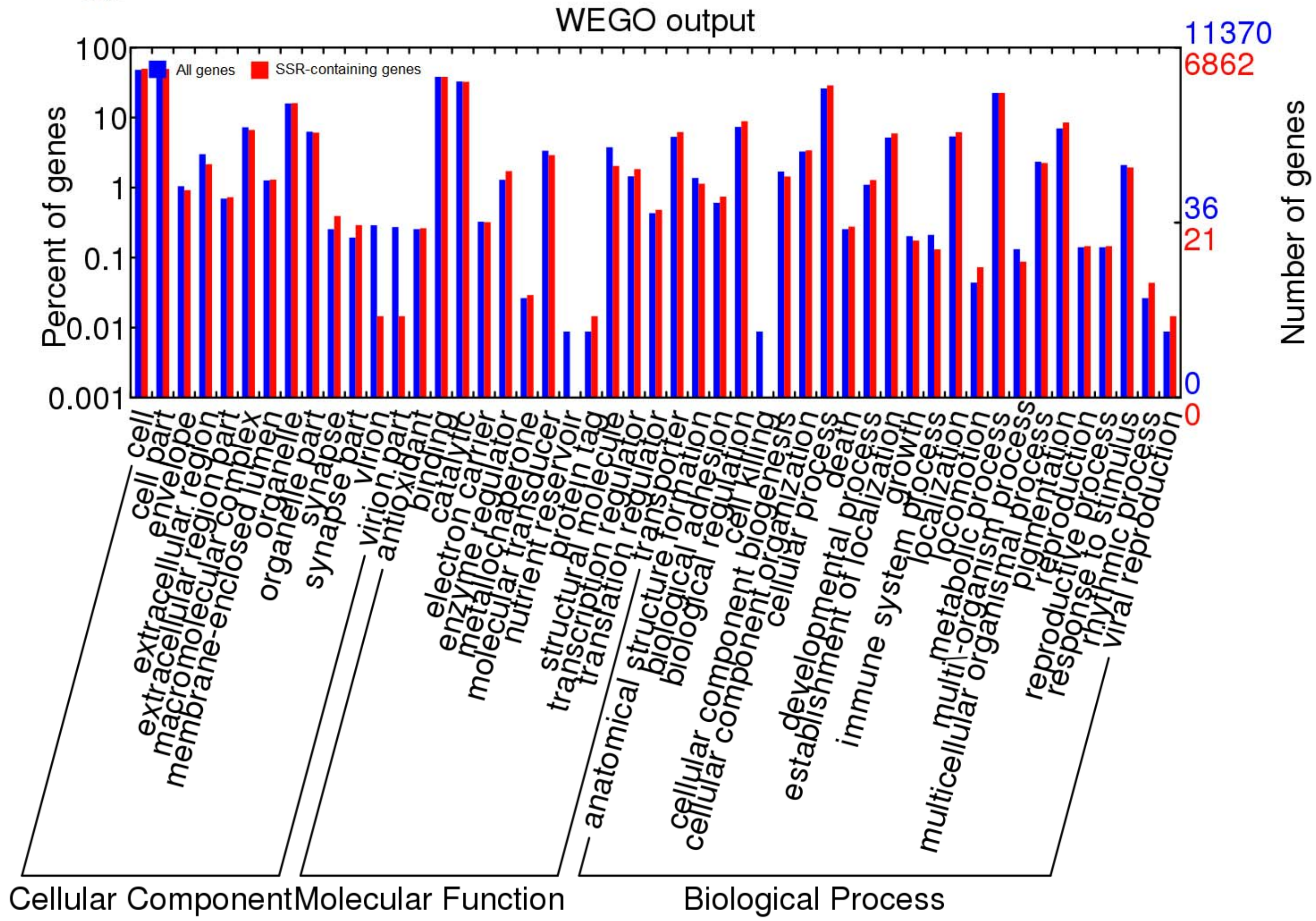

Ae. albopictus

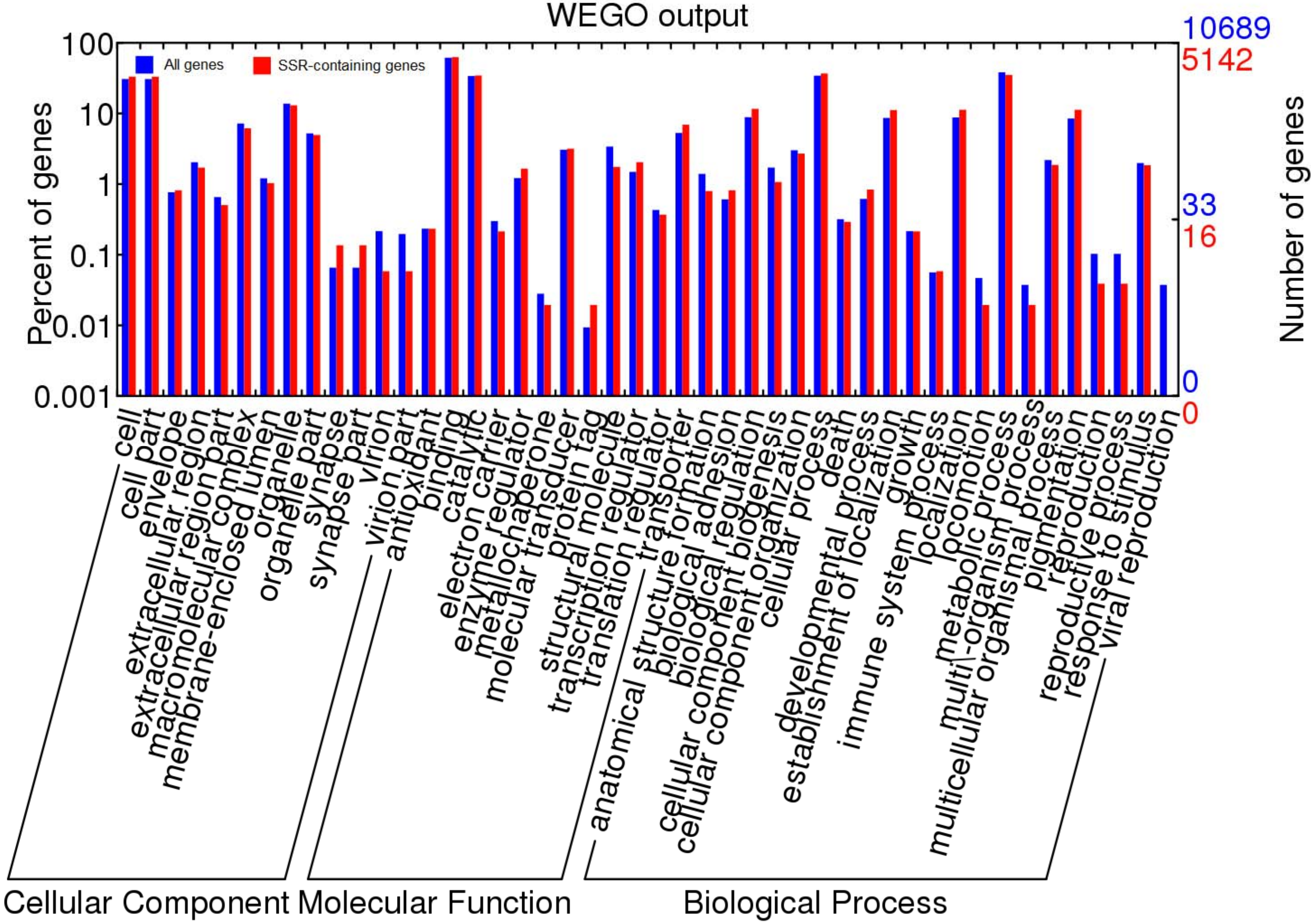

An. albimanus

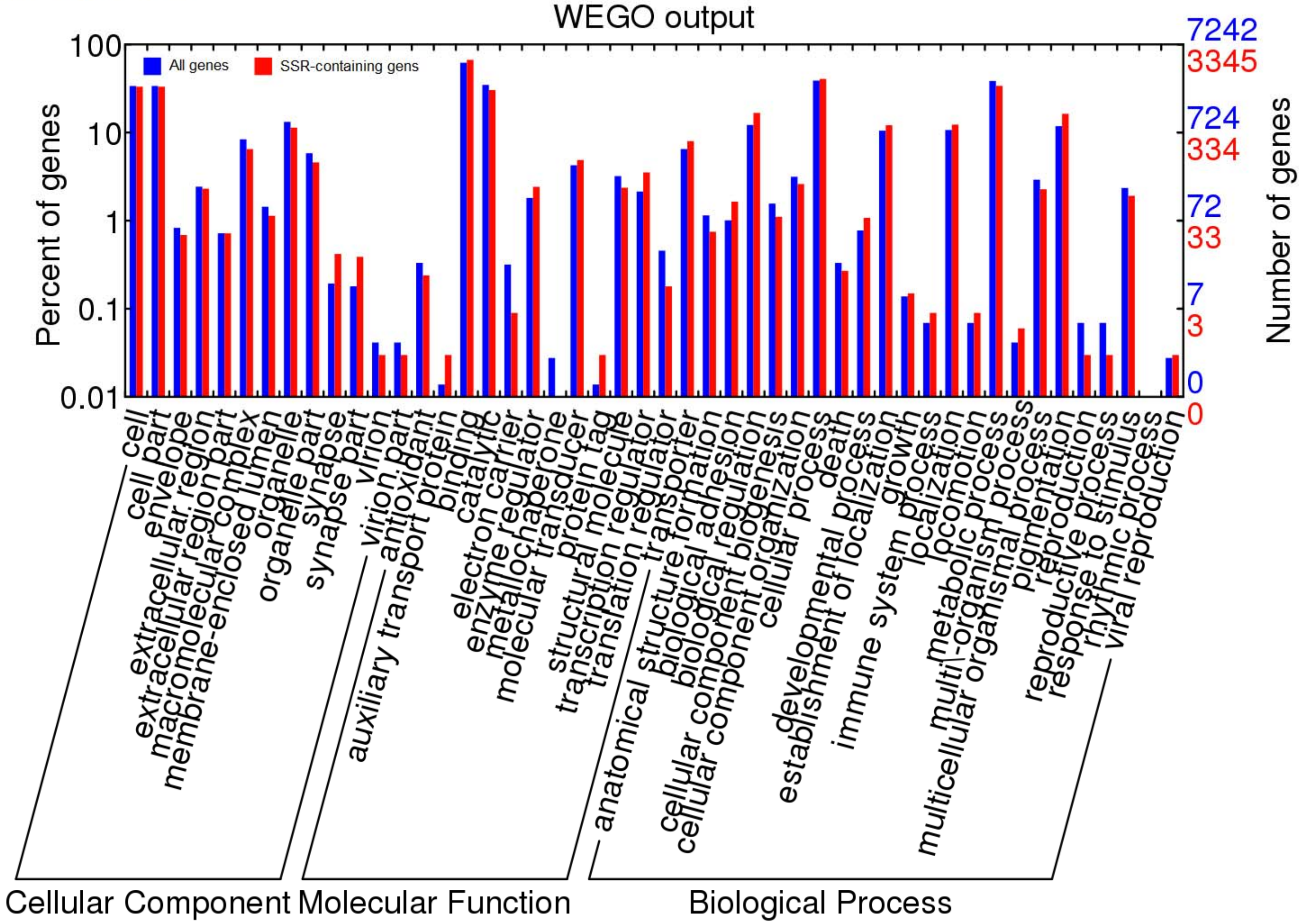

An. arabiensis

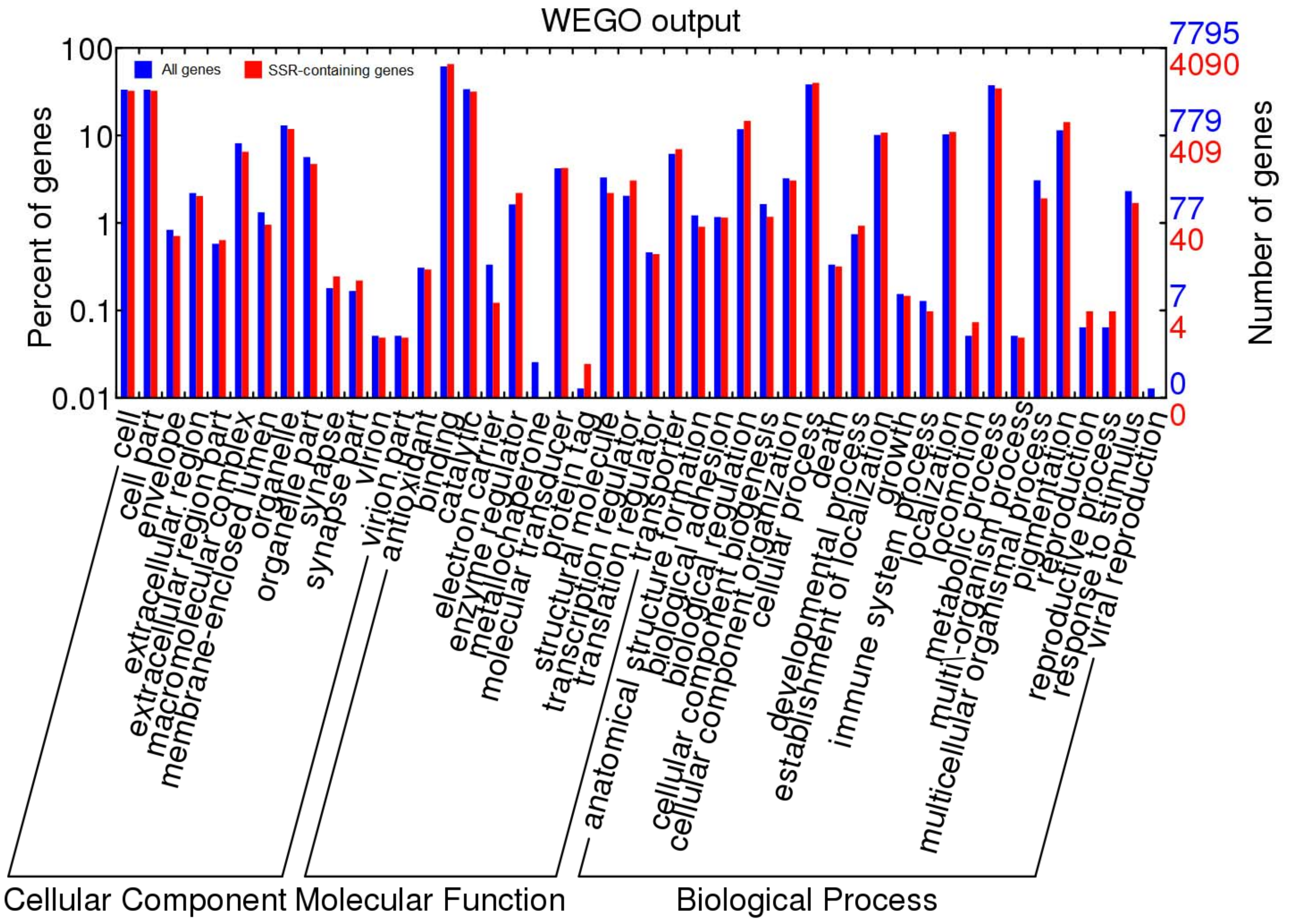

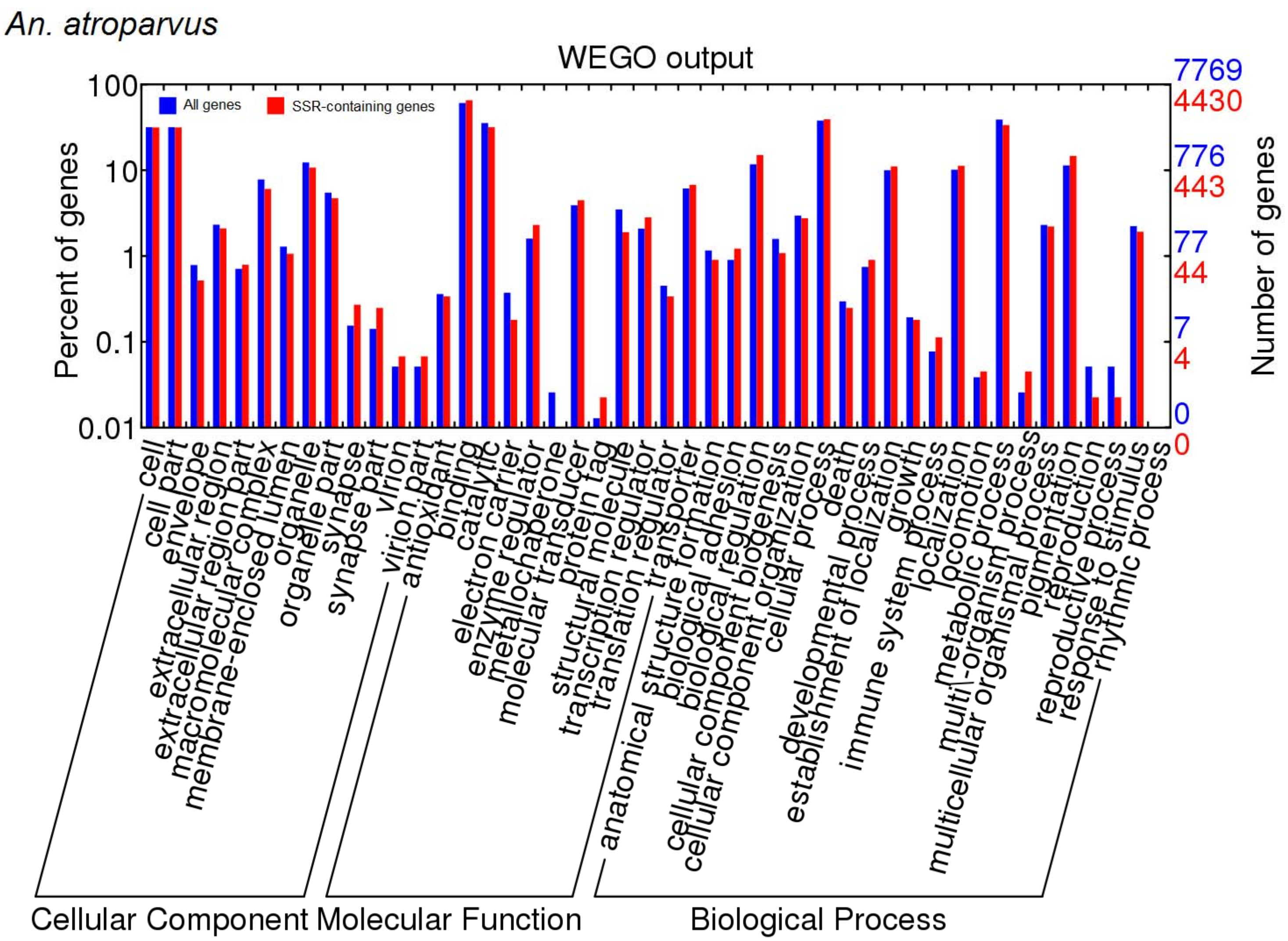

An. atroparvus
WEGO output
All genes
SSR-containing genes
Percent of genes
Number of genes
100
10
1
0.1
0.01
7769
4430
776
443
77
44
7
4
0
0
cell
cell part
envelope
extracellular region
extracellular region part
macromolecular complex
membrane-enclosed lumen
organelle
organelle part
synapse
synapse part
virion
virion part
antioxidant
binding
catalytic
electron carrier
enzyme regulator
metallochaperone
molecular transducer
protein tag
structural molecule
transcription regulator
translation regulator
transporter
anatomical structure formation
biological adhesion
biological regulation
cellular component biogenesis
cellular component organization
cellular process
death
developmental process
establishment of localization
growth
immune system process
localization
locomotion
metabolic process
multi\-organism process
multicellular organismal process
pigmentation
reproduction
reproductive process
response to stimulus
rhythmic process
Cellular Component
Molecular Function
Biological Process

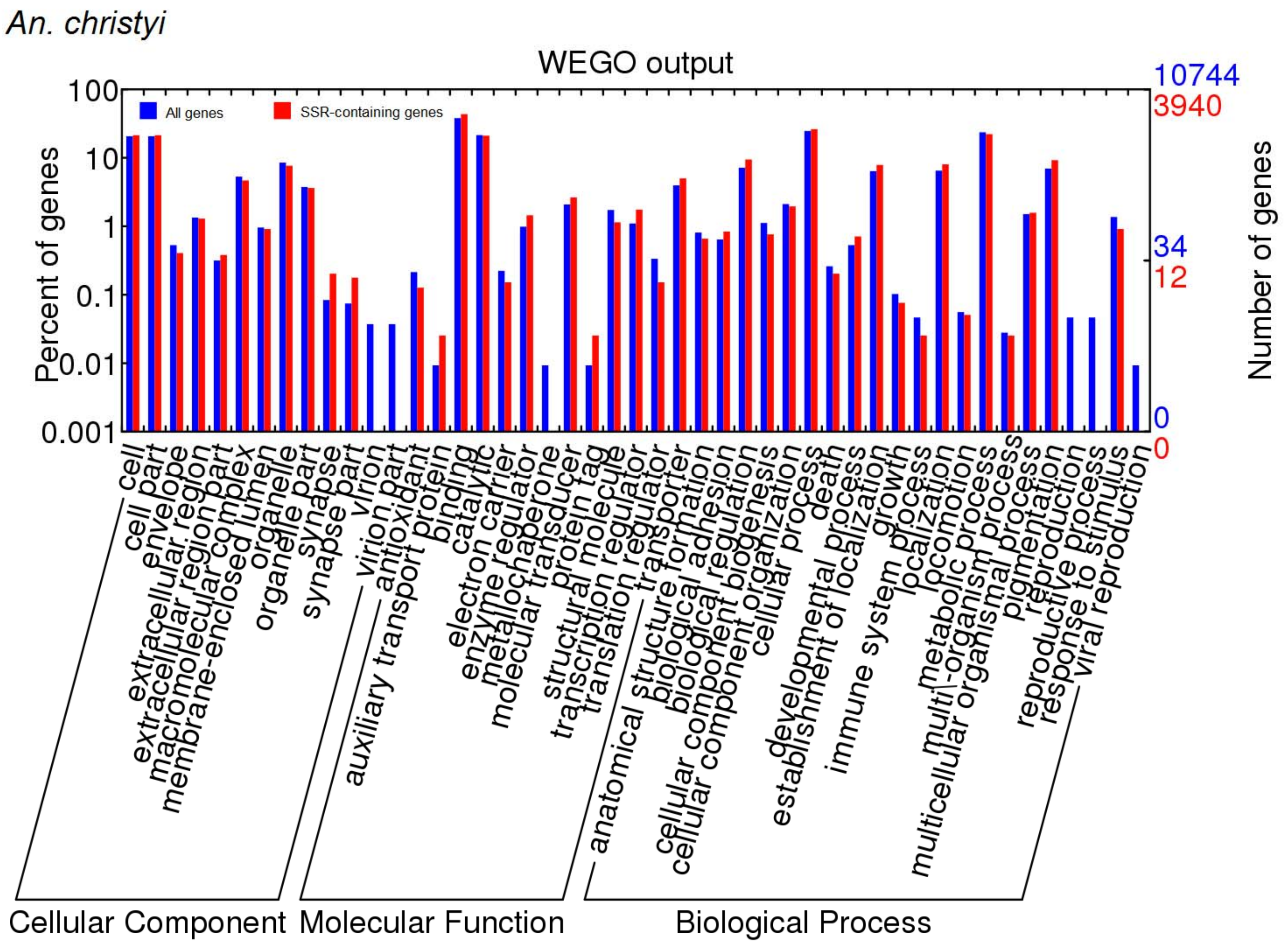
An. christyi
WEGO output
All genes
SSR-containing genes
Percent of genes
Number of genes
100
10
1
0.1
0.01
0.001
10744
3940
34
12
0
0
cell
cell part
envelope
extracellular region
extracellular region part
macromolecular complex
membrane-enclosed lumen
organelle
organelle part
synapse
synapse part
virion
virion part
antioxidant
auxiliary transport protein
binding
catalytic
electron carrier
enzyme regulator
metallochaperone
molecular transducer
protein tag
structural molecule
transcription regulator
translation regulator
transporter
anatomical structure formation
biological adhesion
biological regulation
cellular component biogenesis
cellular component organization
cellular process
death
developmental process
establishment of localization
growth
immune system process
localization
locomotion
metabolic process
multi-organism process
multicellular organismal process
pigmentation
reproduction
reproductive process
response to stimulus
viral reproduction
Cellular Component
Molecular Function
Biological Process

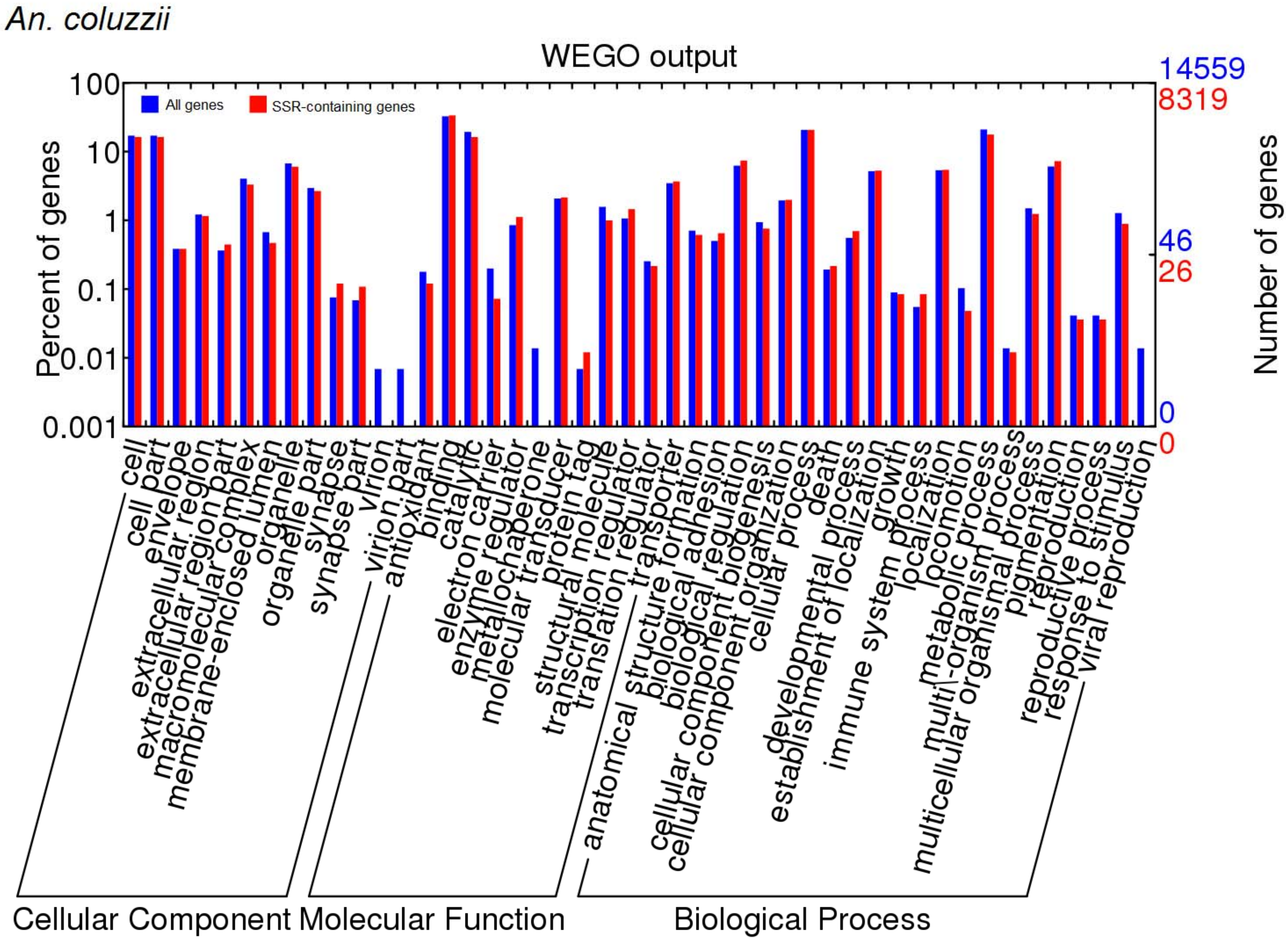

An. coluzzii
WEGO output
All genes
SSR-containing genes
Percent of genes
Number of genes
100
10
1
0.1
0.01
0.001
14559
8319
46
26
0
0
cell
cell part
envelope
extracellular region
extracellular region part
macromolecular complex
membrane-enclosed lumen
organelle
organelle part
synapse
synapse part
virion
virion part
antioxidant
binding
catalytic
electron carrier
enzyme regulator
metallochaperone
molecular transducer
protein tag
structural molecule
transcription regulator
translation regulator
transporter
anatomical structure formation
biological adhesion
biological regulation
cellular component biogenesis
cellular component organization
cellular process
death
developmental process
establishment of localization
growth
immune system process
localization
locomotion
metabolic process
multi\-organism process
multicellular organismal process
pigmentation
reproduction
reproductive process
response to stimulus
viral reproduction
Cellular Component
Molecular Function
Biological Process

An. culicifacies A

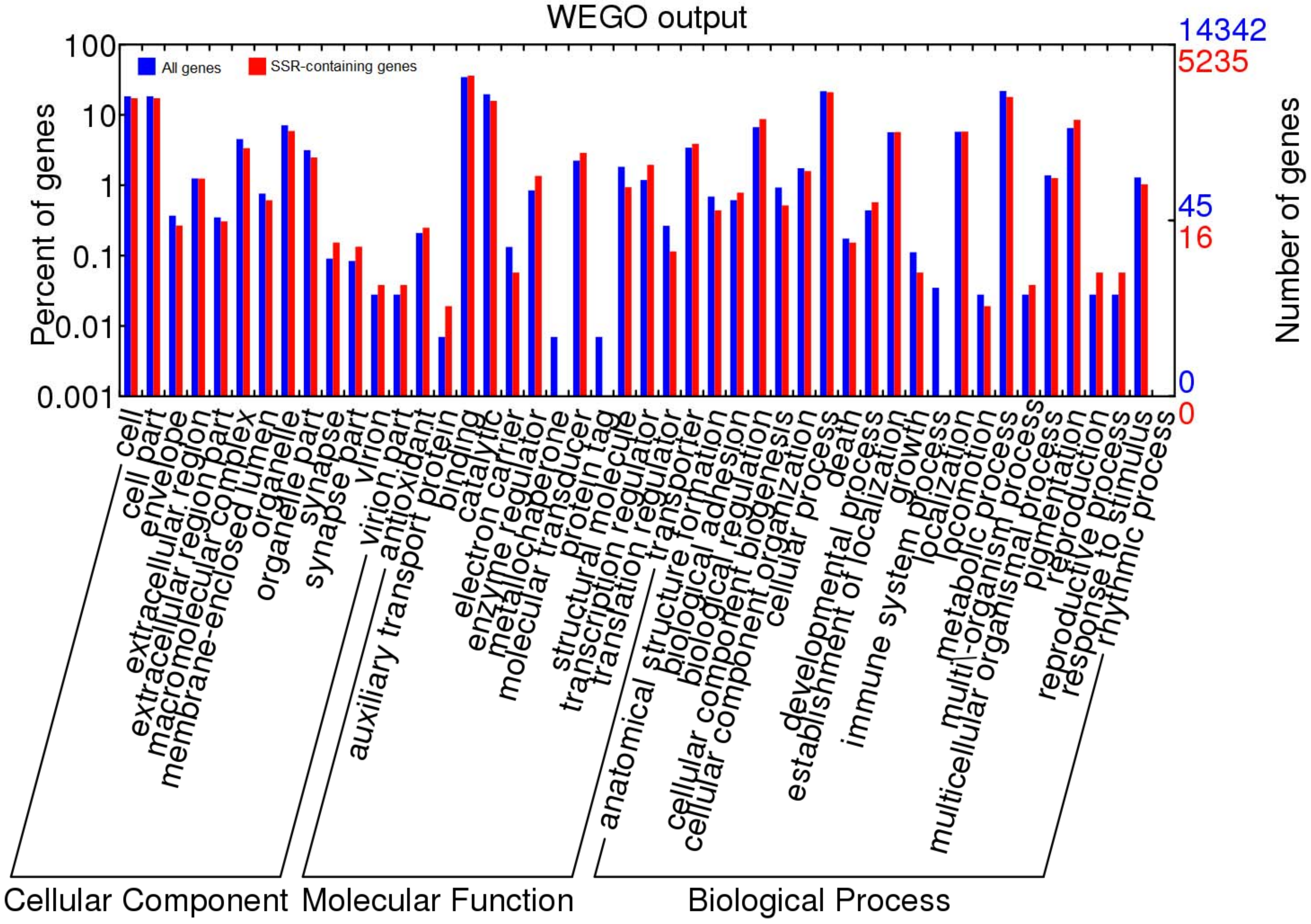

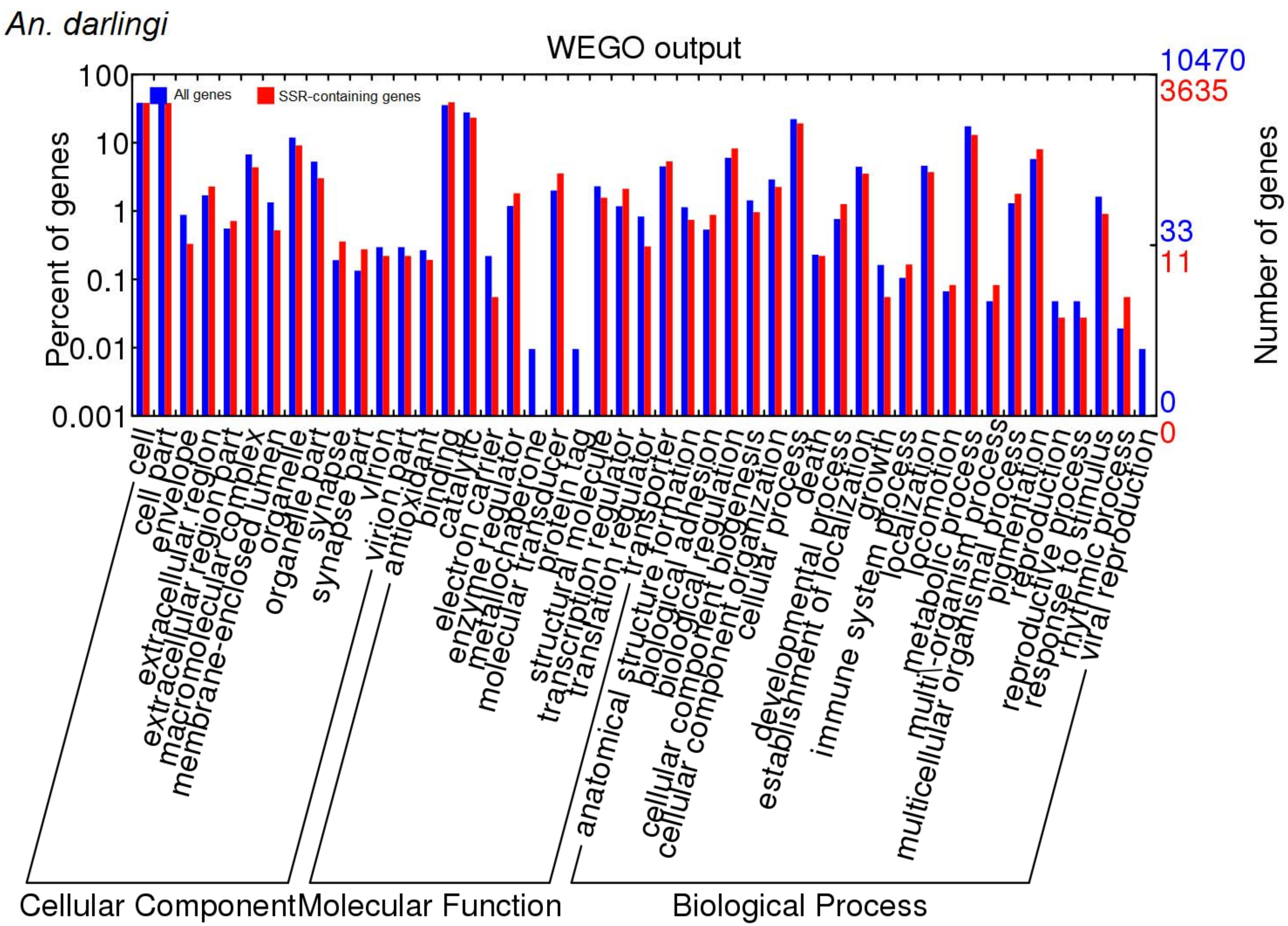
An. darlingi
WEGO output
All genes
SSR-containing genes
Percent of genes
100
10
1
0.1
0.01
0.001
Number of genes
10470
3635
33
11
0
0
cell
cell part
envelope
extracellular region
extracellular region part
macromolecular complex
membrane-enclosed lumen
organelle
organelle part
synapse
synapse part
virion
virion part
antioxidant
binding
catalytic
electron carrier
enzyme regulator
metallochaperone
molecular transducer
protein tag
structural molecule
transcription regulator
translation regulator
transporter
anatomical structure formation
biological adhesion
biological regulation
cellular component biogenesis
cellular component organization
cellular process
death
developmental process
establishment of localization
growth
immune system process
localization
locomotion
metabolic process
multi-organism process
multicellular organismal process
pigmentation
reproduction
reproductive process
response to stimulus
rhythmic process
viral reproduction
Cellular Component
Molecular Function
Biological Process

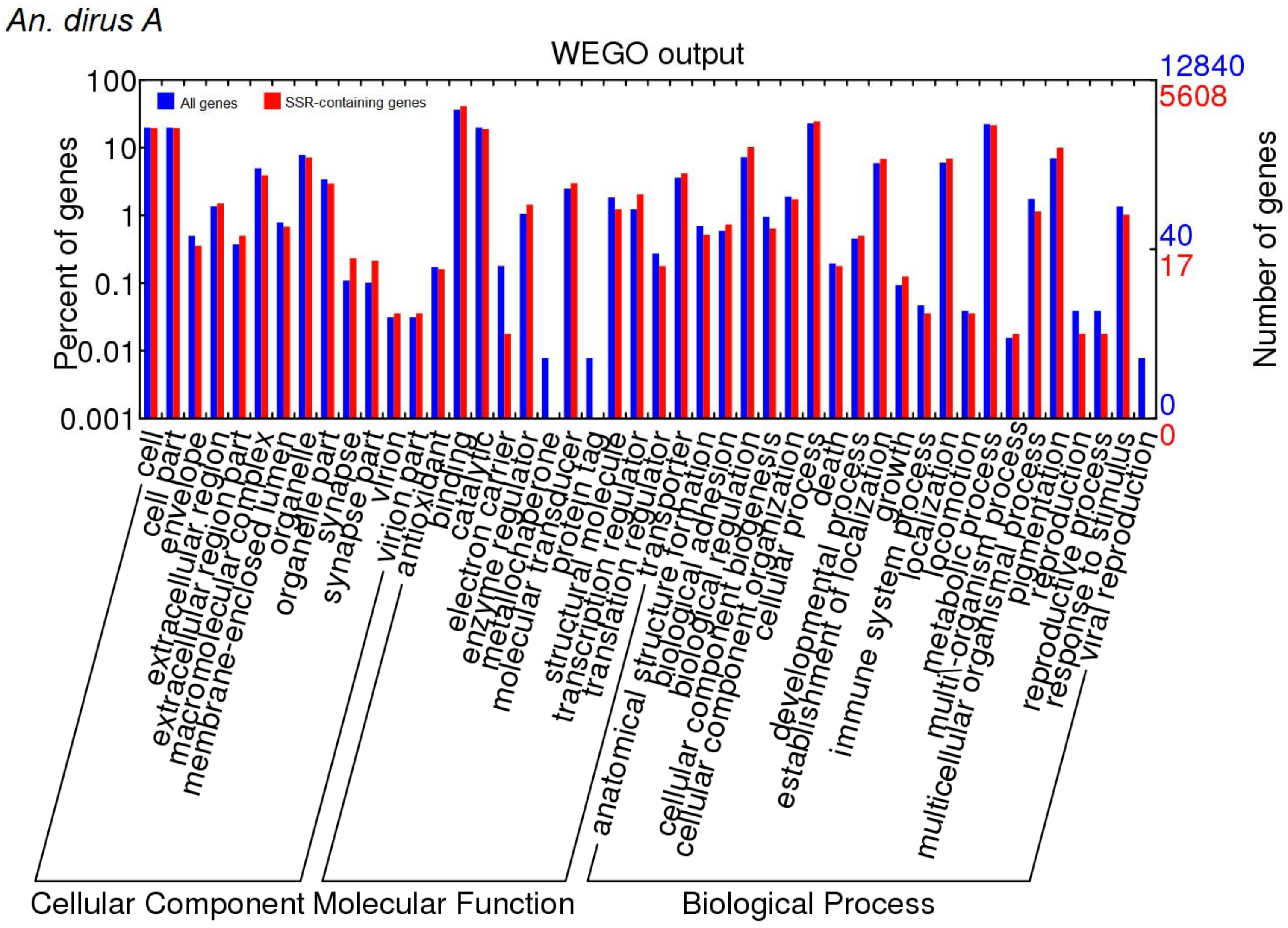

An. dirus A
WEGO output
All genes
SSR-containing genes
Percent of genes
Number of genes
100
10
1
0.1
0.01
0.001
12840
5608
40
17
0
0
cell
cell part
envelope
extracellular region
extracellular region part
macromolecular complex
membrane-enclosed lumen
organelle
organelle part
synapse
synapse part
virion
virion part
antioxidant
binding
catalytic
electron carrier
enzyme regulator
metallochaperone
molecular transducer
protein tag
structural molecule
transcription regulator
translation regulator
transporter
anatomical structure formation
biological adhesion
biological regulation
cellular component biogenesis
cellular component organization
cellular process
death
developmental process
establishment of localization
growth
immune system process
localization
locomotion
metabolic process
multi-organism process
multicellular organismal process
pigmentation
reproduction
reproductive process
response to stimulus
viral reproduction
Cellular Component
Molecular Function
Biological Process

An. epiroticus

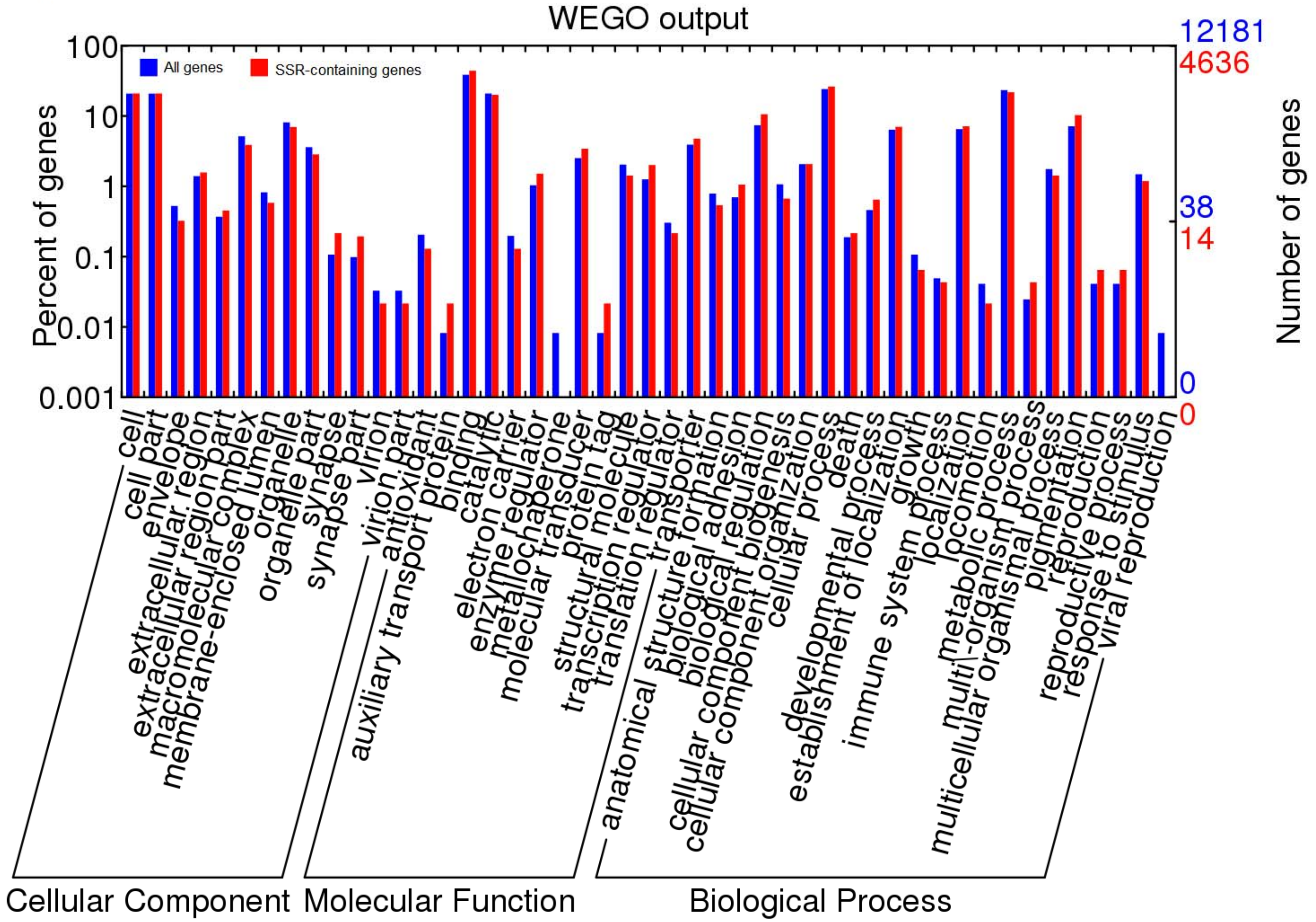

An. farauti

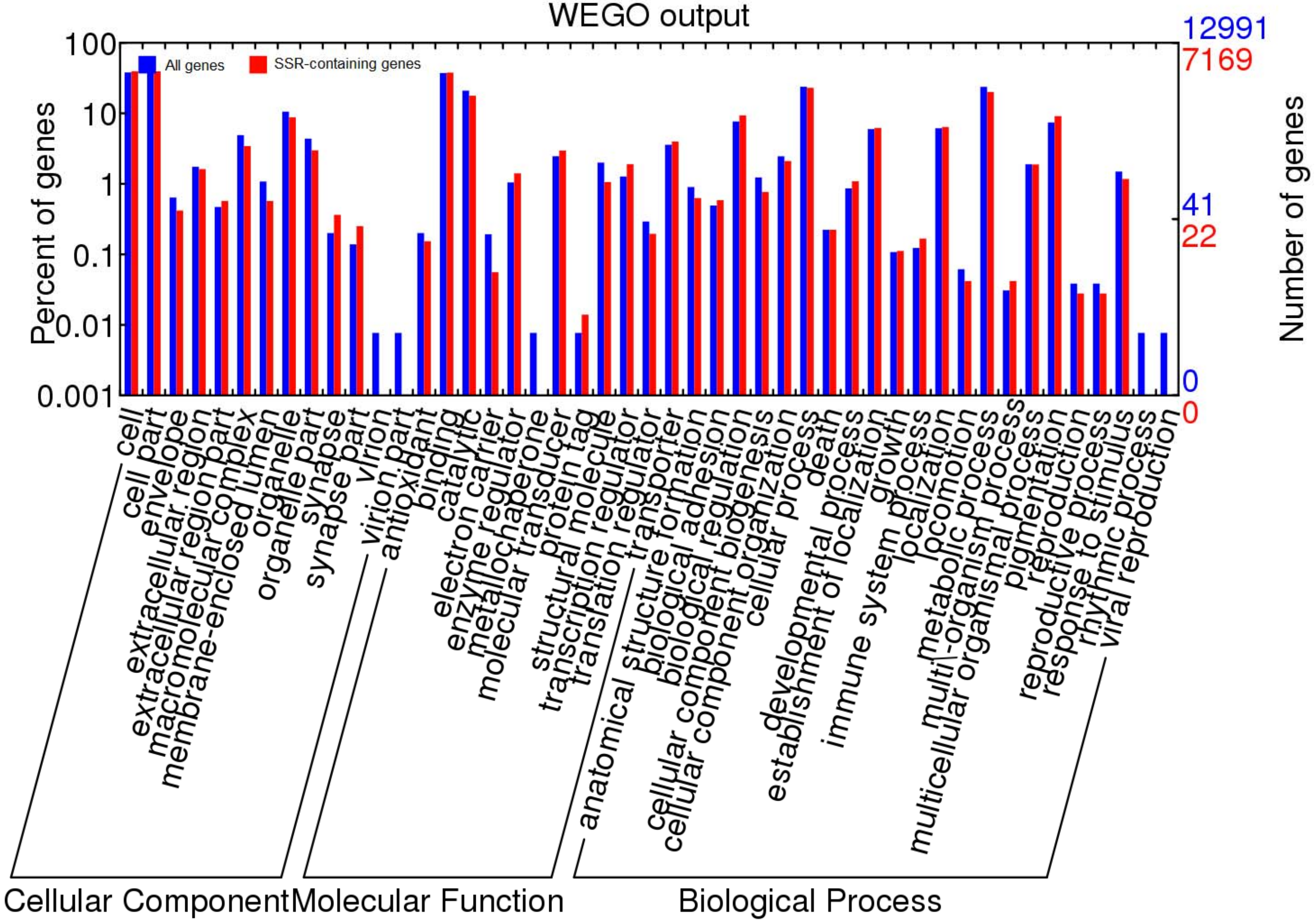

WEGO output
All genes
SSR-containing genes
Percent of genes
100
10
1
0.1
0.01
0.001
Number of genes
12991
7169
41
22
0
0
cell
cell part
envelope
extracellular region
extracellular region part
macromolecular complex
membrane-enclosed lumen
organelle
organelle part
synapse
synapse part
virion
virion part
antioxidant
binding
catalytic
electron carrier
enzyme regulator
metallochaperone
molecular transducer
protein tag
structural molecule
transcription regulator
translation regulator
transporter
anatomical structure formation
biological adhesion
biological regulation
cellular component biogenesis
cellular component organization
cellular process
death
developmental process
establishment of localization
growth
immune system process
localization
locomotion
metabolic process
multi-organism process
multicellular organismal process
pigmentation
reproduction
reproductive process
response to stimulus
rhythmic process
viral reproduction
Cellular Component
Molecular Function
Biological Process

An. funestus

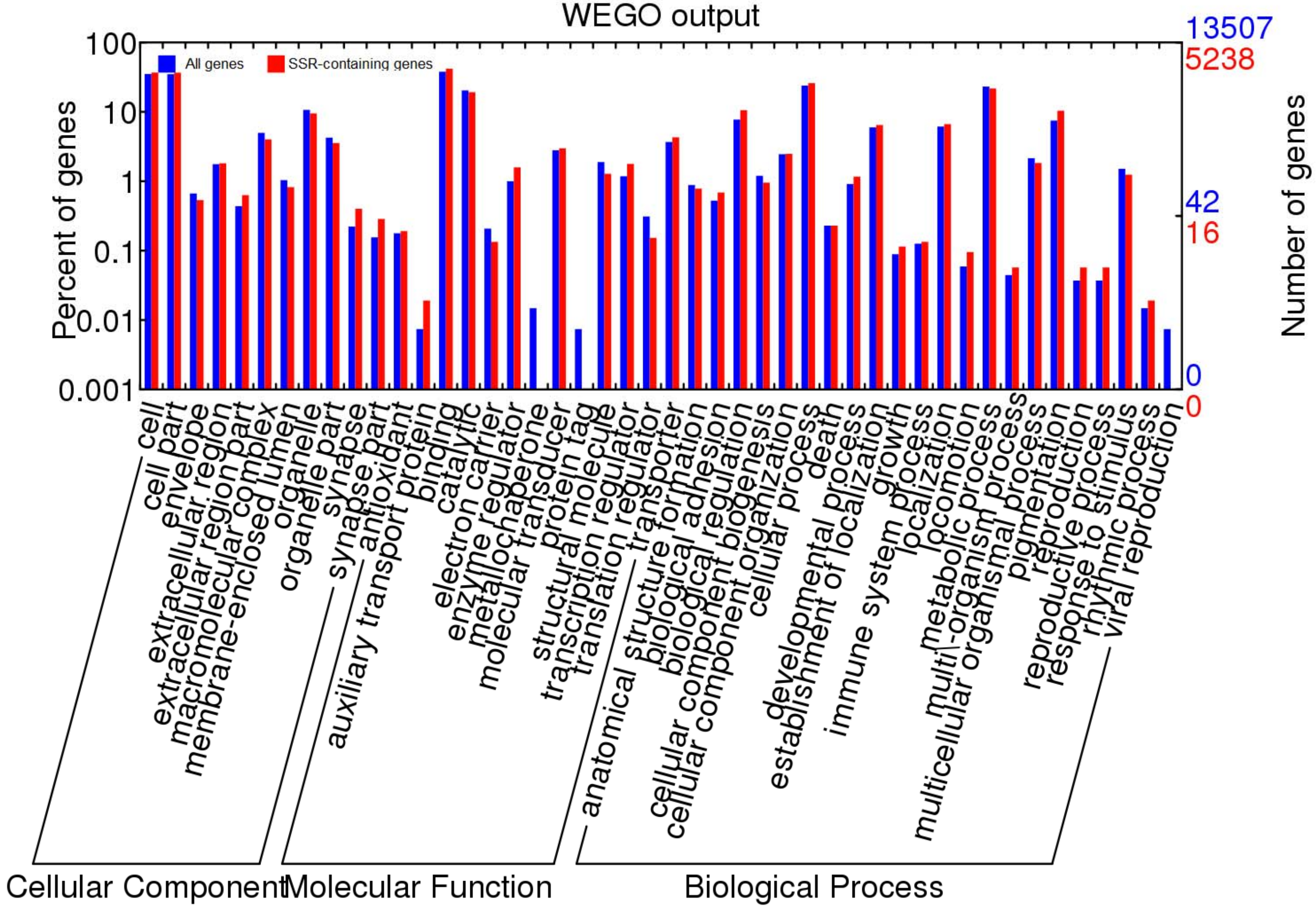

WEGO output
All genes
SSR-containing genes
Percent of genes
Number of genes
100
10
1
0.1
0.01
0.001
13507
5238
42
16
0
0
cell
cell part
envelope
extracellular region
extracellular region part
macromolecular complex
membrane-enclosed lumen
organelle
organelle part
synapse
synapse part
antioxidant
auxiliary transport protein
binding
catalytic
electron carrier
enzyme regulator
metallochaperone
molecular transducer
protein tag
structural molecule
transcription regulator
translation regulator
transporter
anatomical structure formation
biological adhesion
biological regulation
cellular component biogenesis
cellular component organization
cellular process
death
developmental process
establishment of localization
growth
immune system process
localization
locomotion
metabolic process
multi-organism process
multicellular organismal process
pigmentation
reproduction
reproductive process
response to stimulus
rhythmic process
viral reproduction
Cellular Component
Molecular Function
Biological Process

An. gambiae

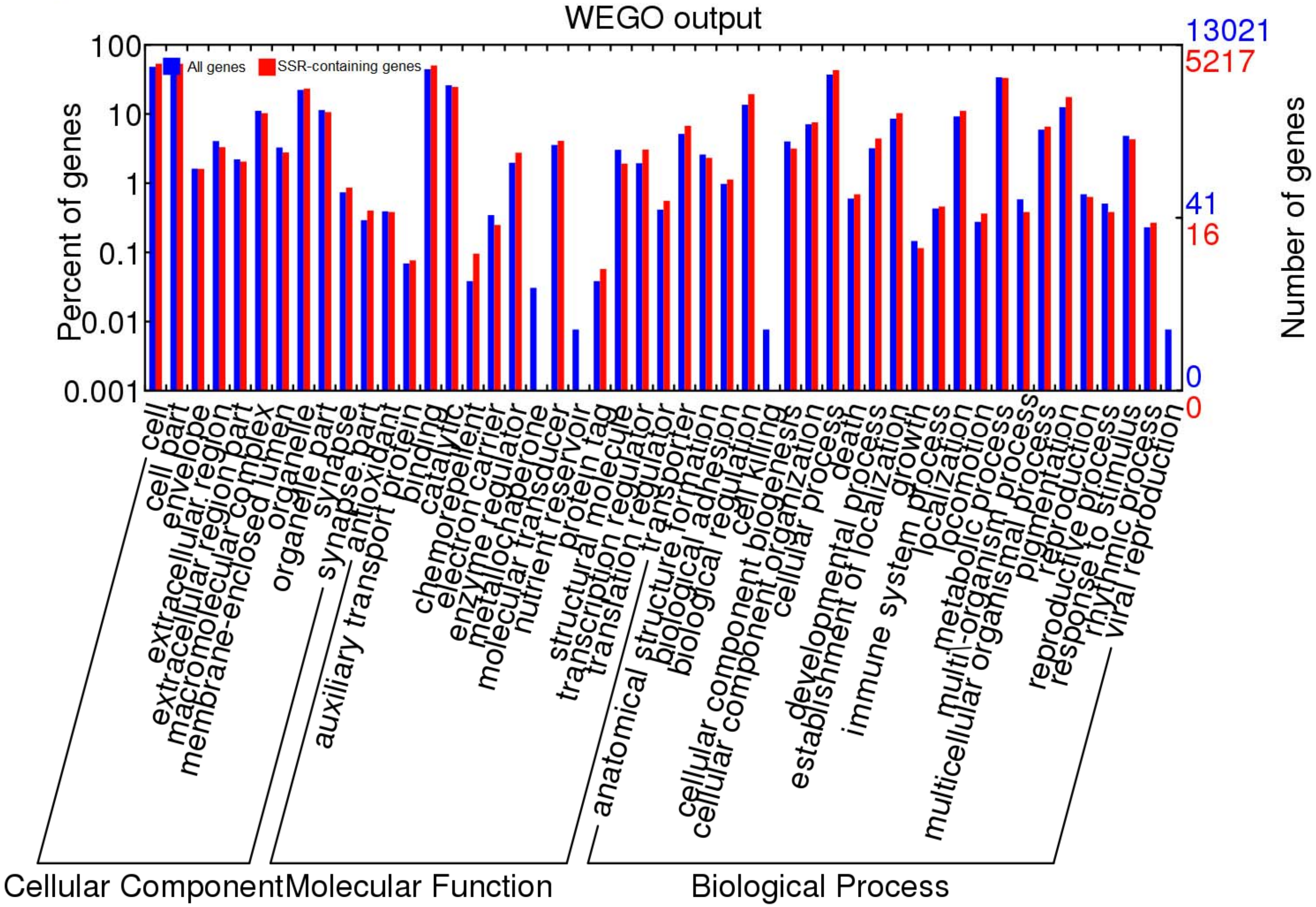

WEGO output
All genes
SSR-containing genes
Percent of genes
Number of genes
100
10
1
0.1
0.01
0.001
13021
5217
41
16
0
0
cell
cell part
envelope
extracellular region
extracellular region part
macromolecular complex
membrane-enclosed lumen
organelle
organelle part
synapse
synapse part
antioxidant
auxiliary transport protein
binding
catalytic
chemorepellent
electron carrier
enzyme regulator
metallochaperone
molecular transducer
nutrient reservoir
protein tag
structural molecule
transcription regulator
translation regulator
transporter
anatomical structure formation
biological adhesion
biological regulation
cell killing
cellular component biogenesis
cellular component organization
cellular process
death
developmental process
establishment of localization
growth
immune system process
localization
locomotion
metabolic process
multi-organism process
multicellular organismal process
pigmentation
reproduction
reproductive process
response to stimulus
rhythmic process
viral reproduction
Cellular Component
Molecular Function
Biological Process

An. maculatus

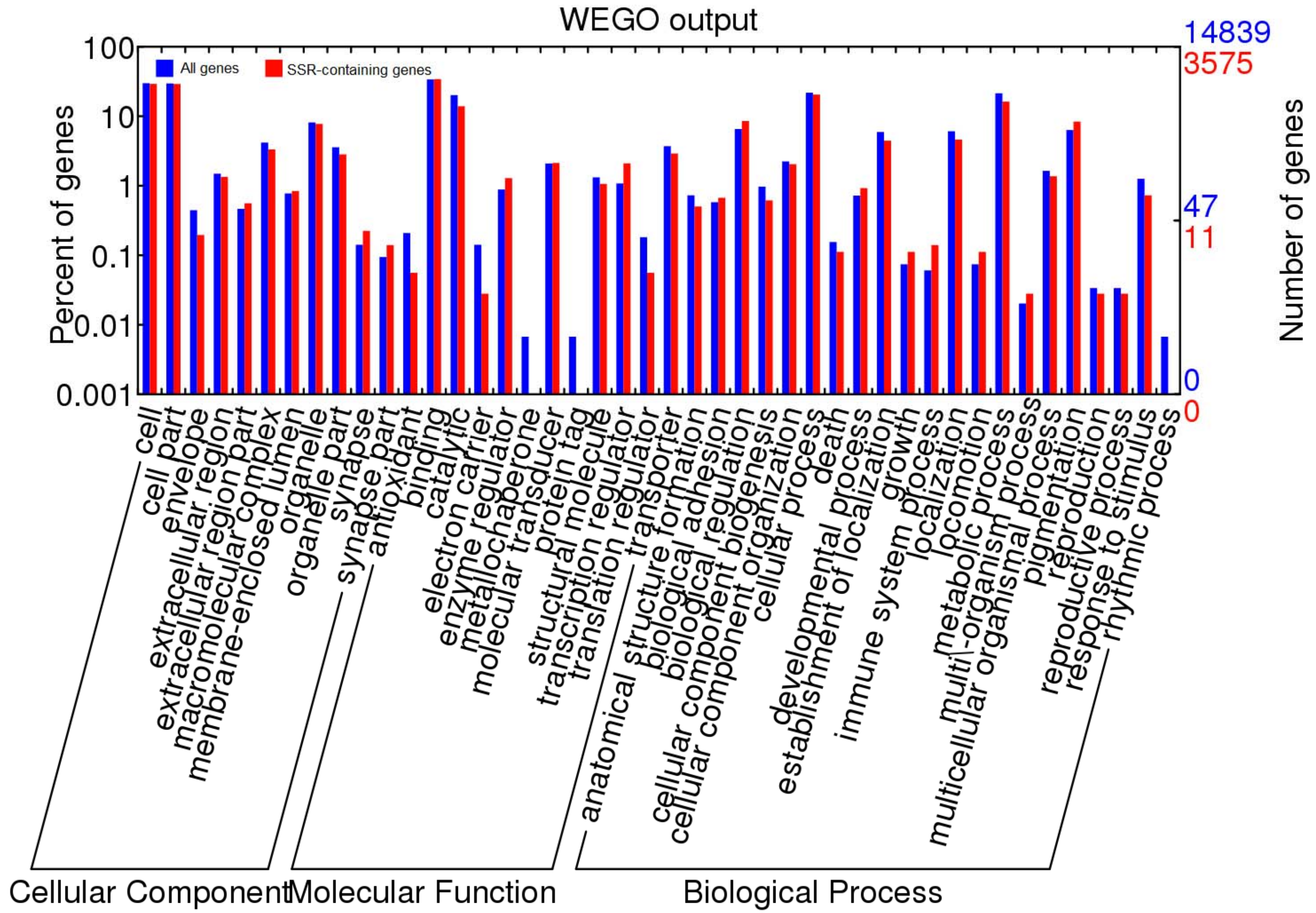

An. melas

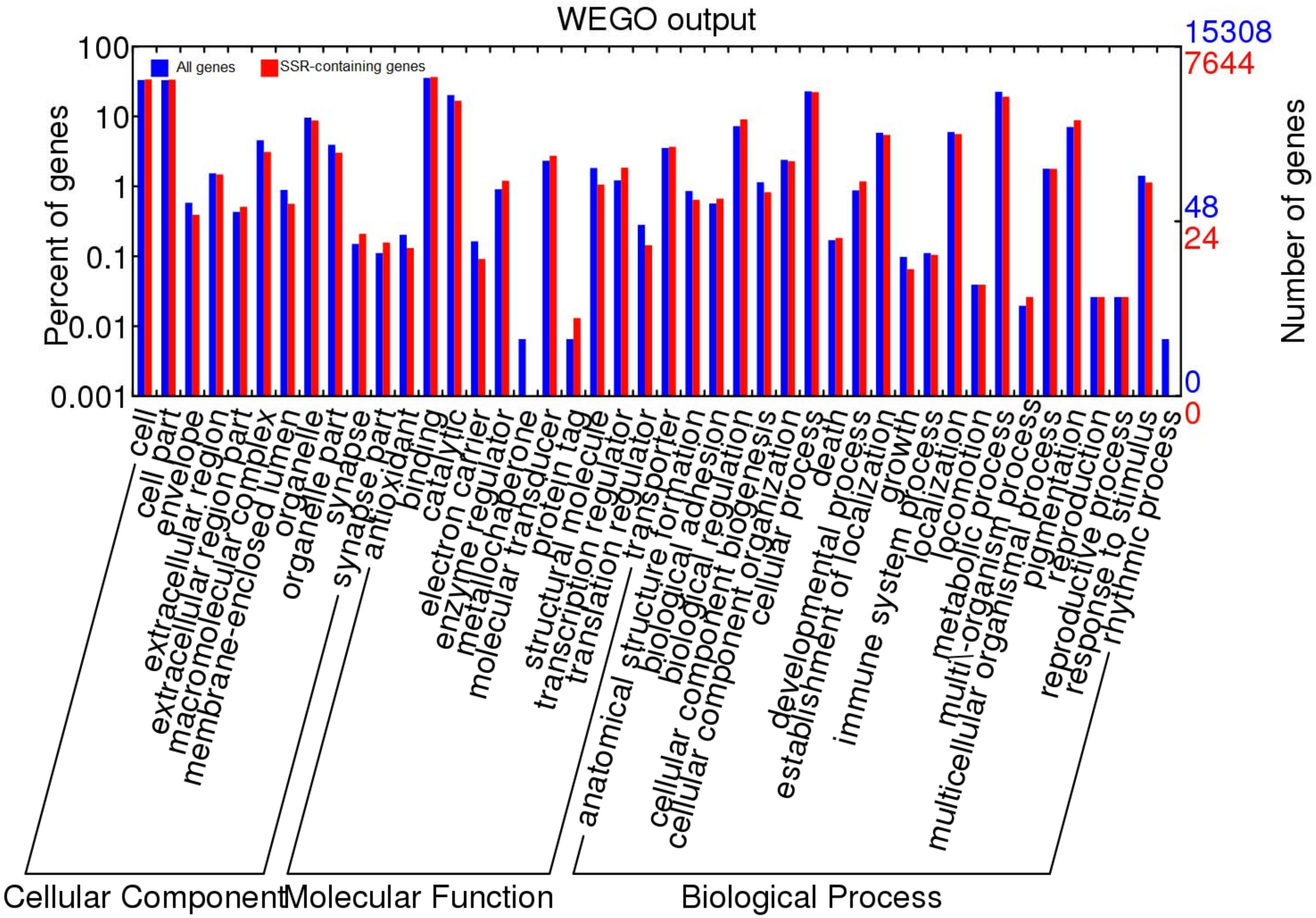
WEGO output
All genes
SSR-containing genes
Percent of genes
Number of genes
100
10
1
0.1
0.01
0.001
15308
7644
48
24
0
0
cell
cell part
envelope
extracellular region
extracellular region part
macromolecular complex
membrane-enclosed lumen
organelle
organelle part
synapse
synapse part
antioxidant
binding
catalytic
electron carrier
enzyme regulator
metallochaperone
molecular transducer
protein tag
structural molecule
transcription regulator
translation regulator
transporter
anatomical structure formation
biological adhesion
biological regulation
cellular component biogenesis
cellular component organization
cellular process
death
developmental process
establishment of localization
growth
immune system process
localization
locomotion
metabolic process
multi-organism process
multicellular organismal process
pigmentation
reproduction
reproductive process
response to stimulus
rhythmic process
Cellular Component
Molecular Function
Biological Process

An. merus

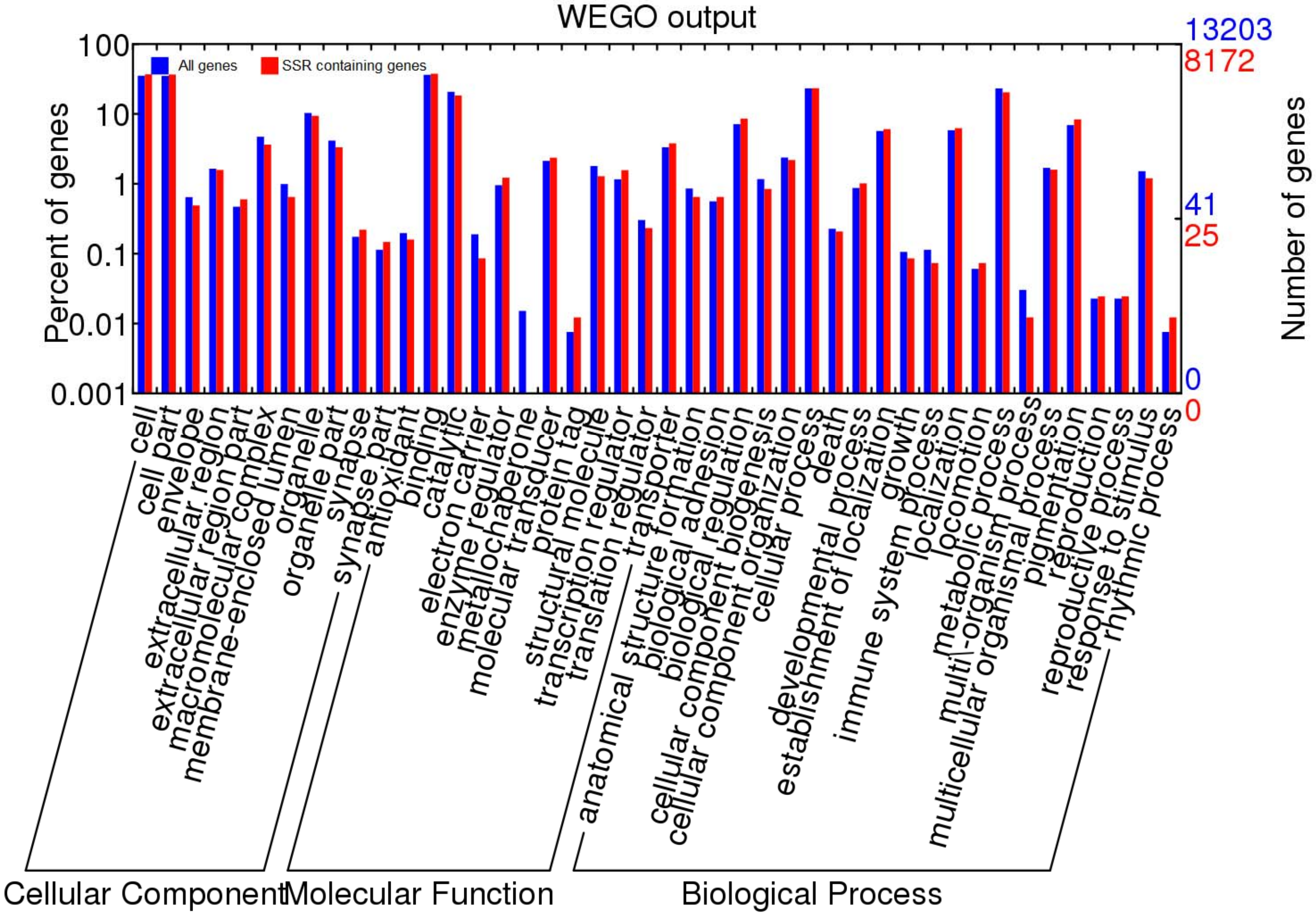
WEGO output
All genes
SSR containing genes
Percent of genes
100
10
1
0.1
0.01
0.001
13203
8172
41
25
0
0
Number of genes
cell
cell part
envelope
extracellular region
extracellular region part
macromolecular complex
membrane-enclosed lumen
organelle
organelle part
synapse
synapse part
antioxidant
binding
catalytic
electron carrier
enzyme regulator
metallochaperone
molecular transducer
protein tag
structural molecule
transcription regulator
translation regulator
transporter
anatomical structure formation
biological adhesion
biological regulation
cellular component biogenesis
cellular component organization
cellular process
death
developmental process
establishment of localization
growth
immune system process
localization
locomotion
metabolic process
multi\-organism process
multicellular organismal process
pigmentation
reproduction
reproductive process
response to stimulus
rhythmic process
Cellular Component
Molecular Function
Biological Process

An. minimus

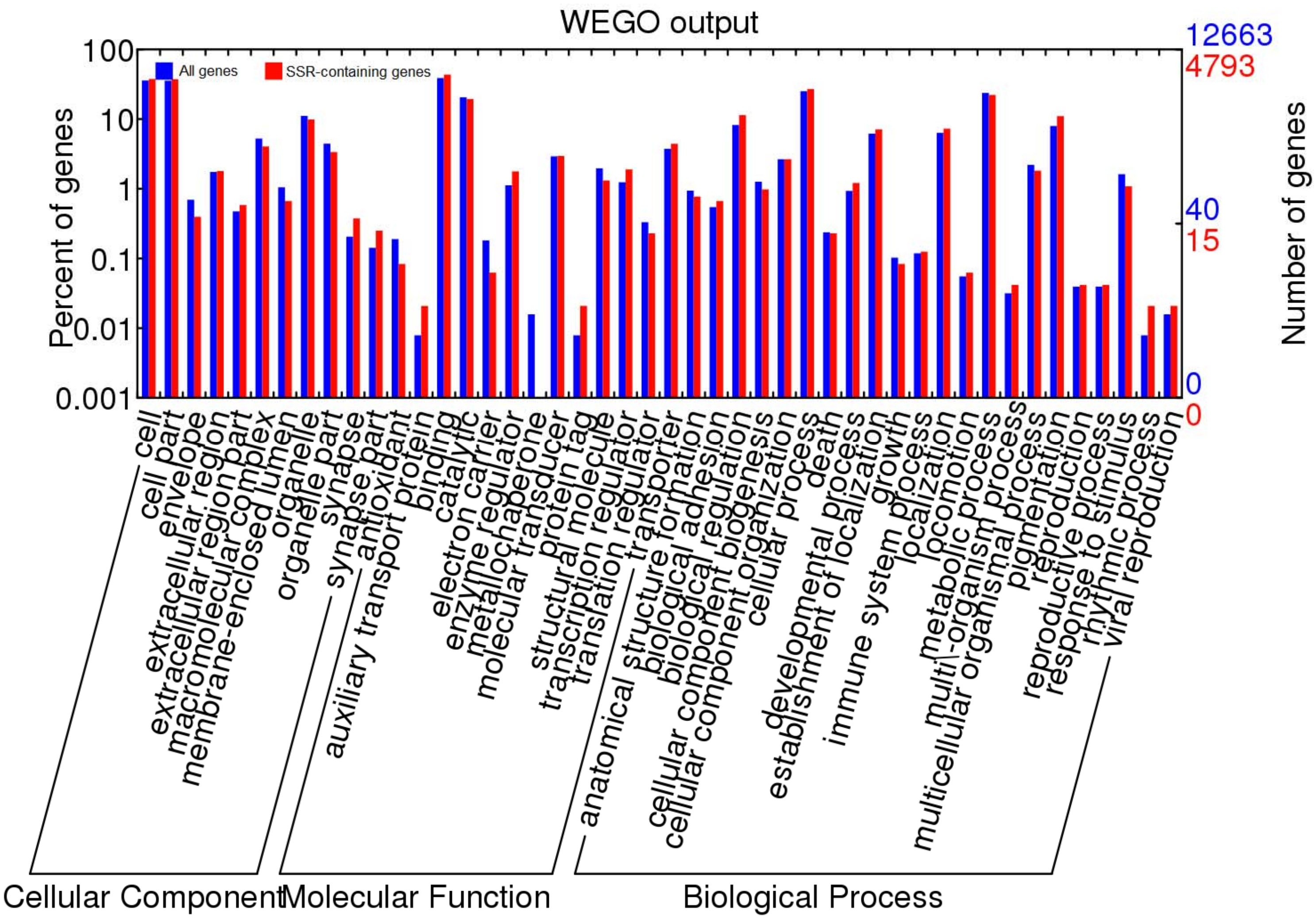

WEGO output
All genes
SSR-containing genes
Percent of genes
Number of genes
100
10
1
0.1
0.01
0.001
12663
4793
40
15
0
0
cell
cell part
envelope
extracellular region
extracellular region part
macromolecular complex
membrane-enclosed lumen
organelle
organelle part
synapse
synapse part
antioxidant
auxiliary transport protein
binding
catalytic
electron carrier
enzyme regulator
metallochaperone
molecular transducer
protein tag
structural molecule
transcription regulator
translation regulator
transporter
anatomical structure formation
biological adhesion
biological regulation
cellular component biogenesis
cellular component organization
cellular process
death
developmental process
establishment of localization
growth
immune system process
localization
locomotion
metabolic process
multi-organism process
multicellular organismal process
pigmentation
reproduction
reproductive process
response to stimulus
rhythmic process
viral reproduction
Cellular Component
Molecular Function
Biological Process

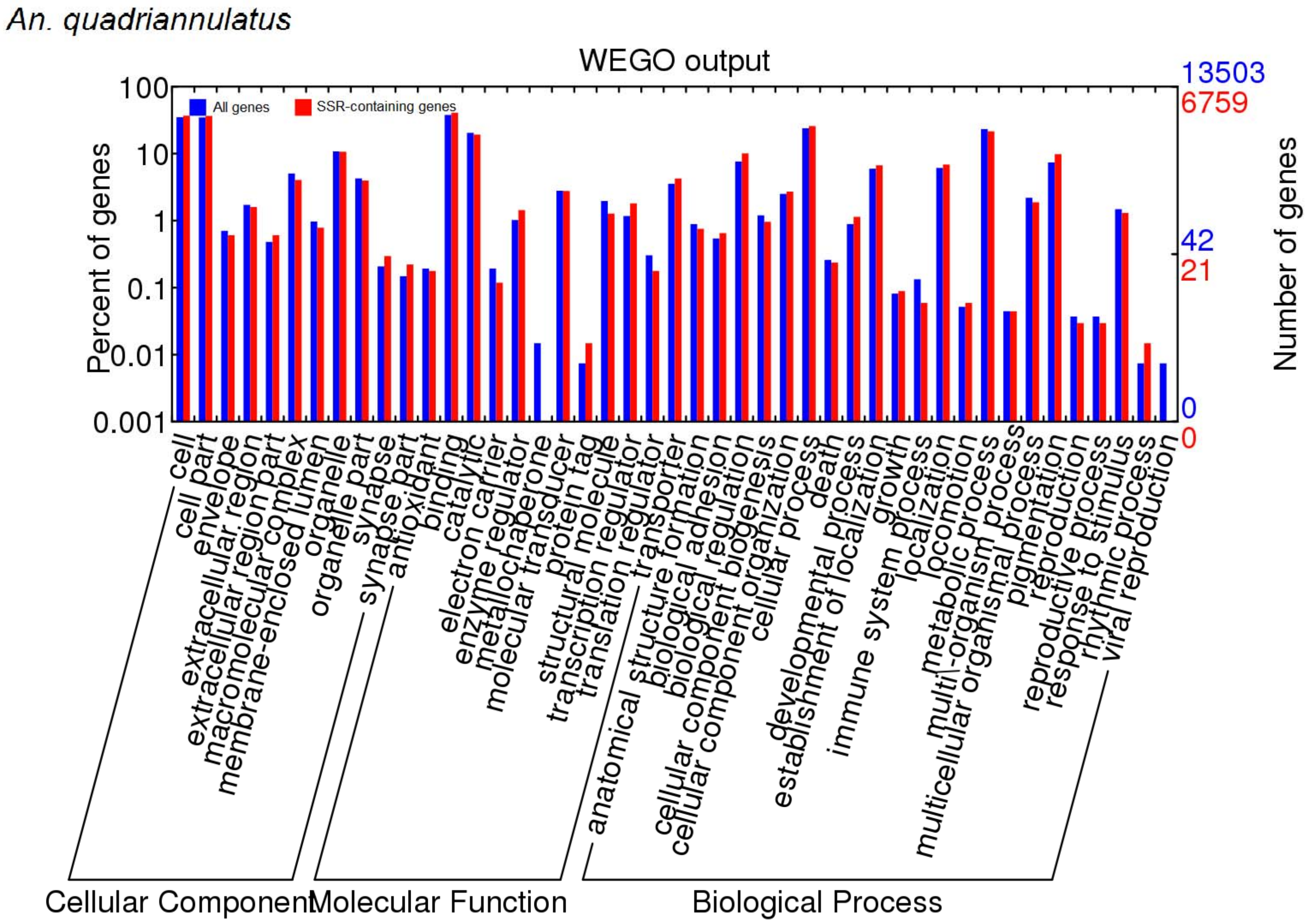
An. quadriannulatus
WEGO output
All genes
SSR-containing genes
Percent of genes
Number of genes
100
10
1
0.1
0.01
0.001
13503
6759
42
21
0
0
cell
cell part
envelope
extracellular region
extracellular region part
macromolecular complex
membrane-enclosed lumen
organelle
organelle part
synapse
synapse part
antioxidant
binding
catalytic
electron carrier
enzyme regulator
metallochaperone
molecular transducer
protein tag
structural molecule
transcription regulator
translation regulator
transporter
anatomical structure formation
biological adhesion
biological regulation
cellular component biogenesis
cellular component organization
cellular process
death
developmental process
establishment of localization
growth
immune system process
localization
locomotion
metabolic process
multi-organism process
multicellular organismal process
pigmentation
reproduction
reproductive process
response to stimulus
rhythmic process
viral reproduction
Cellular Component
Molecular Function
Biological Process

An. sinensis

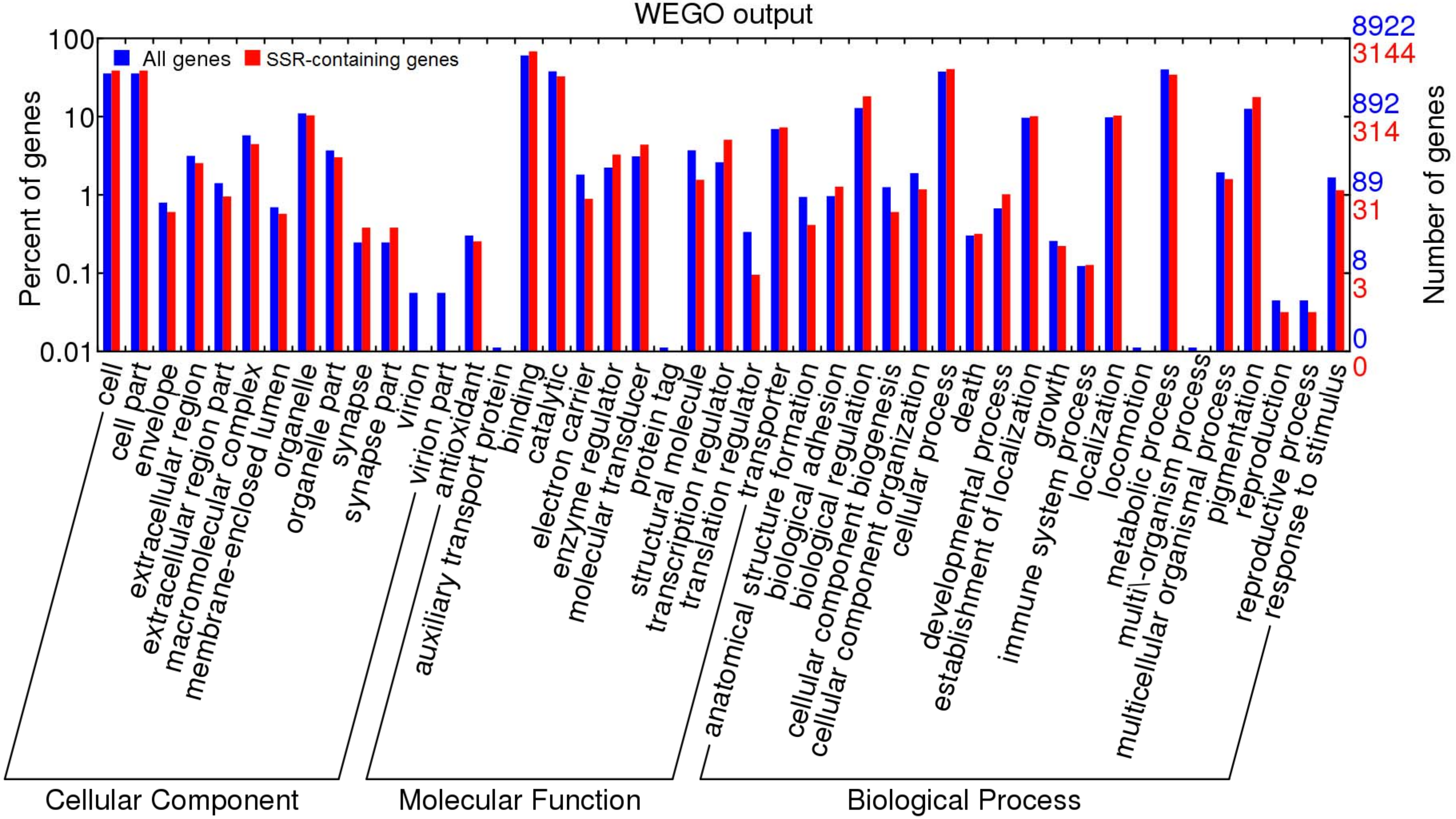
WEGO output
All genes
SSR-containing genes
Percent of genes
Number of genes
100
10
1
0.1
0.01
8922
3144
892
314
89
31
8
3
0
0
cell
cell part
envelope
extracellular region
extracellular region part
macromolecular complex
membrane-enclosed lumen
organelle
organelle part
synapse
synapse part
virion
virion part
antioxidant
auxiliary transport protein
binding
catalytic
electron carrier
enzyme regulator
molecular transducer
protein tag
structural molecule
transcription regulator
translation regulator
transporter
anatomical structure formation
biological adhesion
biological regulation
cellular component biogenesis
cellular component organization
cellular process
death
developmental process
establishment of localization
growth
immune system process
localization
locomotion
metabolic process
multi\-organism process
multicellular organismal process
pigmentation
reproduction
reproductive process
response to stimulus
Cellular Component
Molecular Function
Biological Process

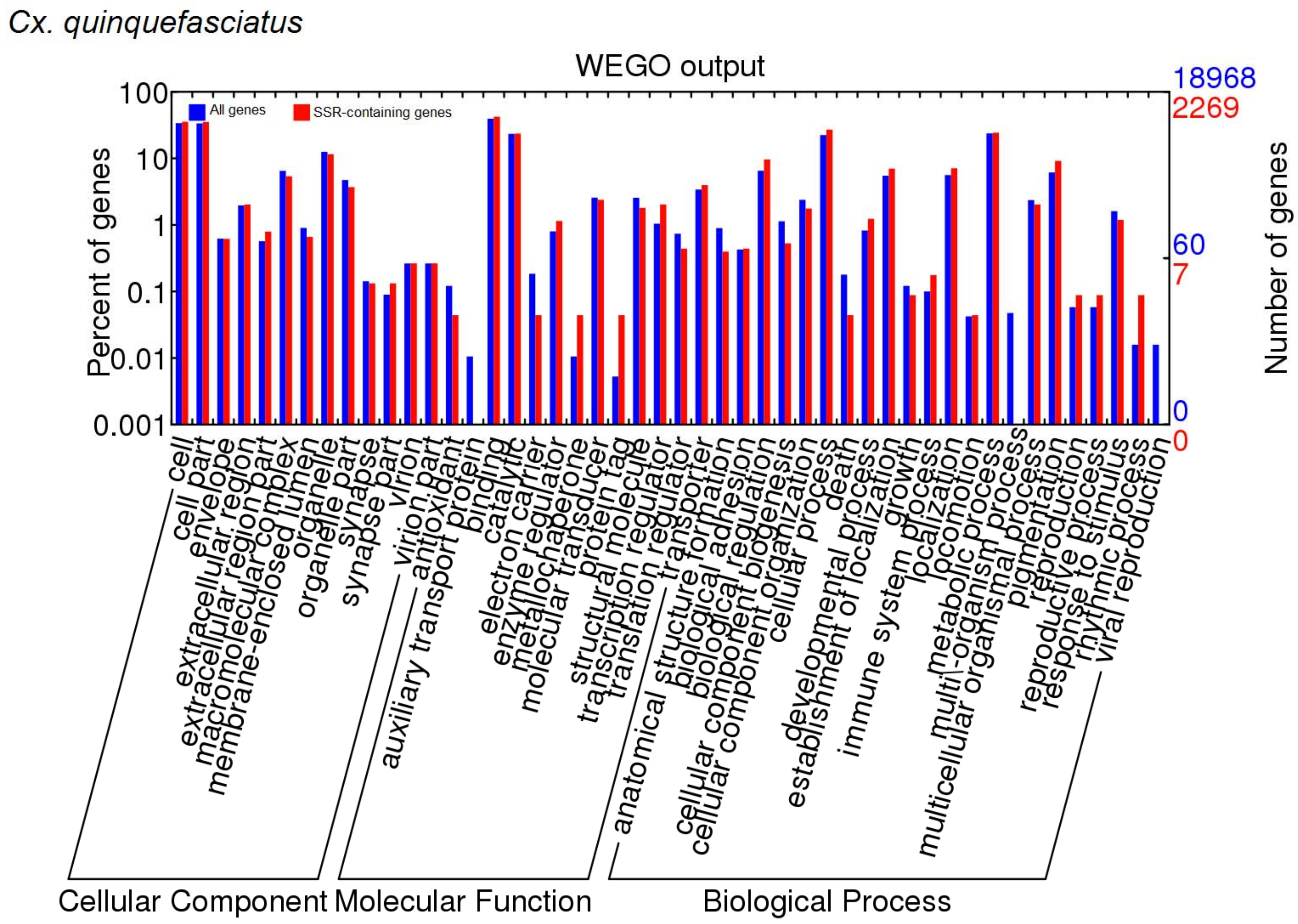
Cx. quinquefasciatus
WEGO output
All genes
SSR-containing genes
Percent of genes
Number of genes
100
10
1
0.1
0.01
0.001
18968
2269
60
7
0
0
cell
cell part
envelope
extracellular region
extracellular region part
macromolecular complex
membrane-enclosed lumen
organelle
organelle part
synapse
synapse part
virion
virion part
antioxidant
auxiliary transport protein
binding
catalytic
electron carrier
enzyme regulator
metallochaperone
molecular transducer
protein tag
structural molecule
transcription regulator
translation regulator
transporter
anatomical structure formation
biological adhesion
biological regulation
cellular component biogenesis
cellular component organization
cellular process
death
developmental process
establishment of localization
growth
immune system process
localization
locomotion
metabolic process
multi\-organism process
multicellular organismal process
pigmentation
reproduction
reproductive process
response to stimulus
rhythmic process
viral reproduction
Cellular Component
Molecular Function
Biological Process
