# Supplementary material for: Comparative analyses of simple sequence repeats (SSRs) in 23 mosquito species genomes: Identification, characterization and distribution (Diptera: Culicidae)
Source: Insect Sci. 2018 Apr 6;26(4):607–19. doi: 10.1111/1744-7917.12577 (PMC7379697; doi:10.1111/1744-7917.12577)
Supplement: Supplementary file 2 — Table S1. The most frequent SSR motifs in the 23 mosquito species and D. melanogaster genomes. [file INS-26-607-s002.pdf]

Table S1 The most frequent SSR motifs in the 23 mosquito species and *D. melanogaster* genomes.

	Mononucleotide		Dinucleotide			
D. melanogaster	A/T (91.71%)	C/G (8.29%)	AC/GT (54.96%)	AT/AT (31.41%)	AG/CT (13.59%)	CG/CG (0.04%)
Ae. albopictus	A/T (91.92%)	C/G (8.08%)	AC/GT (49.67%)	AG/CT (33.87%)	AT/AT (15.26%)	CG/CG (0.04%)
Ae. aegypti	A/T (95.49%)	C/G (4.51%)	AT/AT (41.07%)	AG/CT (33.87%)	AC/GT (28.16%)	CG/CG (0.65%)
Cx. quinquefasciatus	A/T (91.44%)	C/G (8.56%)	AC/GT (67.05%)	AG/CT (24.74%)	AT/AT (5.89%)	CG/CG (2.32%)
An. darlingi	A/T (70.88%)	C/G (29.12%)	AC/GT (58.77%)	AG/CT (37.18%)	CG/CG (2.97%)	AT/AT (1.08%)
An. albimanus	A/T (77.48%)	C/G (22.52%)	AG/CT (47.95%)	AC/GT (46.20%)	CG/CG (3.94%)	AT/AT (1.92%)
An. sinensis	A/T (86.22%)	C/G (13.78%)	AC/GT (75.23%)	AG/CT (19.58%)	AT/AT (3.07%)	CG/CG (2.12%)
An. atroparvus	A/T (86.92%)	C/G (13.08%)	AC/GT (63.74%)	AG/CT (30.88%)	AT/AT (3.51%)	CG/CG (1.87%)
An. nili	A/T (97.73%)	C/G (2.27%)	AC/GT (47.15%)	AG/CT (43.05%)	AT/AT (6.75%)	CG/CG (3.05%)
An. dirus A	A/T (79.91%)	C/G (20.09%)	AC/GT (58.79%)	AG/CT (34.82%)	CG/CG (4.57%)	AT/AT (1.82%)
An. farauti	A/T (88.80%)	C/G (11.20%)	AC/GT (62.23%)	AG/CT (32.88%)	AT/AT (2.67%)	CG/CG (2.22%)
An. funestus	A/T (92.43%)	C/G (7.57%)	AC/GT (63.15%)	AG/CT (33.09%)	AT/AT (2.45%)	CG/CG (1.31%)
An. mininus A	A/T (90.05%)	C/G (9.95%)	AC/GT (70.81%)	AG/CT (23.74%)	AT/AT (4.34%)	CG/CG (1.74%)
An. culicifacies A	A/T (93.81%)	C/G (6.19%)	AC/GT (70.18%)	AG/CT (25.61%)	AT/AT (2.95%)	CG/CG (1.25%)
An. maculatus	A/T (93.85%)	C/G (6.15%)	AC/GT (72.45%)	AG/CT (24.40%)	CG/CG (1.74%)	AT/AT (1.41%)
An. stephensi	A/T (80.48%)	C/G (19.52%)	AC/GT (67.08%)	AG/CT (30.39%)	AT/AT (1.40%)	CG/CG (1.13%)
An. epiroticus	A/T (78.67%)	C/G (21.33%)	AC/GT (61.33%)	AG/CT (35.23%)	AT/AT (2.13%)	CG/CG (1.31%)
An. christyi	A/T (89.88%)	C/G (10.12%)	AC/GT (67.27%)	AG/CT (27.92%)	AT/AT (4.08%)	CG/CG (0.74%)
An. melas	A/T (74.17%)	C/G (25.83%)	AC/GT (68.01%)	AG/CT (28.12%)	AT/AT (2.46%)	CG/CG (1.41%)
An. merus	A/T (75.00%)	C/G (25.00%)	AC/GT (67.85%)	AG/CT (28.73%)	CG/CG (1.75%)	AT/AT (1.67%)
An. quadriannulatus	A/T (76.53%)	C/G (23.47%)	AC/GT (69.41%)	AG/CT (27.34%)	CG/CG (1.81%)	AT/AT (1.43%)
An. arabiensis	A/T (76.62%)	C/G (23.38%)	AC/GT (68.90%)	AG/CT (27.71%)	CG/CG (1.82%)	AT/AT (1.58%)
An. gambiase	A/T (75.87%)	C/G (24.13%)	AC/GT (69.20%)	AG/CT (26.56%)	AT/AT (2.49%)	CG/CG (1.76%)
An. coluzzii	A/T (79.95%)	C/G (20.05%)	AC/GT (68.81%)	AG/CT (27.39%)	AT/AT (1.90%)	CG/CG (1.89%)

	Trinucleotide			
D. melanogaster	AGC/GCT (34.96%)	AAT/ATT (19.10%)	AAC/GTT (16.57%)	ATC/GAT (9.44%)
Ae. albopictus	AAG/CTT (30.43%)	ACG/CGT (21.66%)	AAT/ATT (12.30%)	ATC/GAT (12.21%)
Ae. aegypti	AAG/CTT (50.26%)	AAT/ATT (16.10%)	ACG/CGT (9.50%)	ATC/GAT (8.78%)
Cx. quinquefasciatus	AGC/GCT (24.81%)	ACG/CGT (24.52%)	AAC/GTT (15.82%)	AAT/ATT (8.12%)
An. darlingi	AGC/GCT (40.82%)	ACG/CGT (17.93%)	ACC/GGT (12.13%)	ATC/GAT (11.38%)
An. albimanus	AGC/GCT (52.77%)	ACG/CGT (16.25%)	ATC/GAT (11.87%)	ACC/GGT (6.07%)
An. sinensis	AGC/GCT (22.99%)	ATC/GAT (16.67%)	ACG/CGT (15.82%)	ACC/GGT (14.49%)
An. atroparvus	AGC/GCT (35.18%)	ACG/CGT (15.77%)	ATC/GAT (13.24%)	ACC/GGT (8.05%)
An. nili	AGC/GCT (31.39%)	ATC/GAT (17.75%)	ACG/CGT (14.32%)	AAC/GTT (11.01%)
An. dirus A	AGC/GCT (35.80%)	ACC/GGT (13.46%)	ACG/CGT (11.96%)	AAC/GTT (8.82%)
An. farauti	AGC/GCT (36.03%)	ACC/GGT (19.37%)	ACG/CGT (11.27%)	ATC/GAT (10.04%)
An. funestus	AGC/GCT (25.90%)	ATC/GAT (19.97%)	AAC/GTT (13.96%)	AAG/CTT (11.06%)
An. mininus A	AGC/GCT (26.14%)	ATC/GAT (21.72%)	AAG/CTT (12.82%)	ACC/GGT (10.24%)
An. culicifacies A	AGC/GCT (24.65%)	ATC/GAT (19.55%)	AAG/CTT (19.91%)	ACC/GGT (9.98%)
An. maculatus	AGC/GCT (35.25%)	AAG/CTT (12.53%)	ATC/GAT (12.38%)	ACC/GGT (12.33%)
An. stephensi	AGC/GCT (33.65%)	AAG/CTT (17.22%)	ATC/GAT (12.53%)	ACC/GGT (11.60%)
An. epiroticus	AGC/GCT (36.57%)	ATC/GAT (12.46%)	AAG/CTT (10.02%)	ACC/GGT (10.01%)
An. christyi	AGC/GCT (28.86%)	AAC/GTT (16.69%)	ATC/GAT (16.49%)	ACC/GGT (12.08%)
An. melas	AGC/GCT (34.23%)	AAG/CTT (12.80%)	AAC/GTT (10.91%)	AAG/CTT (10.90%)
An. merus	AGC/GCT (33.89%)	AAG/CTT (14.01%)	ACC/GGT (10.03%)	ATC/GAT (9.82%)
An. quadriannulatus	AGC/GCT (34.02%)	AAG/CTT (13.13%)	ACC/GGT (10.59%)	ATC/GAT (10.17%)
An. arabiensis	AGC/GCT (33.71%)	AAG/CTT (13.24%)	ACC/GGT (10.36%)	ATC/GAT (10.34%)
An. gambiase	AGC/GCT (31.03%)	AAG/CTT (14.32%)	AAT/ATT (11.71%)	ATC/GAT (9.59%)
An. coluzzii	AGC/GCT (33.49%)	AAG/CTT (13.95%)	ATC/GAT (10.18%)	ACC/GGT (9.68%)

	Teranucleotide			
D. melanogaster	ACAT/ATGT (16.16%)	ACTG/CAGT (10.89%)	AAAT/ATTT (9.16%)	AAAC/GTTT (9.08%)
Ae. albopictus	AAAT/ATTT (29.49%)	ACAG/CTGT (27.72 %)	ACAT/ATGT (9.45%)	AAGT/ACTT (5.01%)
Ae. aegypti	AAAT/ATTT (64.56%)	ACAT/ATGT (7.80%)	AGAT/ATCT (5.38%)	AAGT/ACTT (4.20%)
Cx. quinquefasciatus	ACAT/ATGT (24.73%)	AAAT/ATTT (23.11%)	AAAC/GTTT (12.25%)	AGGC/CCTG (6.60%)
An. darlingi	AGCC/GGCT (24.98%)	ACCG/CGGT (9.82%)	AACG/CGTT (9.10%)	AACC/GGTT (7.75%)
An. albimanus	AGCC/GGCT (27.67%)	AGCG/CGCT (9.68%)	ACCG/CGGT (8.46%)	ACGC/GCGT (7.20%)
An. sinensis	AAAT/ATTT (19.70%)	AAAC/GTTT (14.84%)	AACG/CGTT (9.47%)	ACGC/GCGT (6.54%)
An. atroparvus	AAAT/ATTT (16.58%)	AAAC/GTTT (15.44%)	AACG/CGTT (9.61%)	ACGC/GCGT (6.98%)
An. nili	ACGC/GCGT (12.57%)	AAAC/GTTT (11.70%)	AACG/CGTT (11.70%)	AAAT/ATTT (7.89%)
An. dirus A	AAAT/ATTT (10.32%)	AGCC/GGCT (9.27%)	AAAC/GTTT (8.80%)	AGGC/GCCT (7.68%)
An. farauti	AAAT/ATTT (21.16%)	AAGT/ACTT (11.08%)	AAAC/GTTT (10.45%)	ACGC/GCGT (7.84%)
An. funestus	AAAT/ATTT (26.88%)	AAAC/GTTT (20.13%)	AAGT/ACTT (5.48%)	ATCC/ATGG (5.22%)
An. mininus A	AAAT/ATTT (30.74%)	AAAC/GTTT (14.64%)	AAGT/ACTT (13.18%)	ATCC/ATGG (5.41%)
An. culicifacies A	AAAT/ATTT (29.98%)	AAAC/GTTT (15.89%)	ATCC/GGAT (7.31%)	AAGT/ACTT (6.36%)
An. maculatus	AAAT/ATTT (17.76%)	AAAC/GTTT (13.51%)	AAGT/ACTT (13.30%)	AGCC/GGCT (5.53%)
An. stephensi	AAAT/ATTT (22.42%)	AGCC/GGCT (12.84%)	AAGT/ACTT (8.22%)	AAAC/GTTT (7.78%)
An. epiroticus	AAAT/ATTT (20.93%)	AAGT/ACTT (12.87%)	AAAC/GTTT (12.73%)	AGCC/GGCT (5.80%)
An. christyi	AAAC/GTTT (20.21%)	AAAT/ATTT (17.11%)	AGCC/GGCT (6.10%)	ATCC/GGAT (5.67%)
An. melas	AAAT/ATTT (25.80%)	AAAC/GTTT (15.13%)	AGCC/GGCT (6.52%)	ACCG/CGGT (5.89%)
An. merus	AAAT/ATTT (26.26%)	AAAC/GTTT (12.98%)	AAGT/ACTT (6.62%)	AGCC/GGCT (6.28%)
An. quadriannulatus	AAAT/ATTT (29.02%)	AAAC/GTTT (13.07%)	AGCC/GGCT (7.20%)	AAGT/ACTT (5.52%)
An. arabiensis	AAAT/ATTT (27.24%)	AAAC/GTTT (13.78%)	AGCC/GGCT (7.14%)	AAGT/ACTT (6.64%)
An. gambiase	AAAT/ATTT (33.22%)	AAAC/GTTT (12.40%)	AGCC/GGCT (6.49%)	AAGT/ACTT (6.39%)
An. coluzzii	AAAT/ATTT (30.09%)	AAAC/GTTT (12.66%)	AGCC/GGCT (7.45%)	AAGT/ACTT (5.57%)

	Pentanucleotide			
D. melanogaster	AAGAT/ATCTT (20.22%)	AATAT/ATATT (19.11%)	AATAG/CTATT (17.62%)	AAGAG/CTCTT (4.71%)
Ae. albopictus	ACTAG/CTAGT (20.07%)	AGCAT/ATGCT (16.98%)	AATCT/AGATT (10.03%)	AAAGC/GCTTT (8.93%)
Ae. aegypti	AAGCT/AGCTT (38.43%)	AGCAT/ATGCT (24.24%)	AATAT/ATATT (10.51%)	AATCT/AGATT (6.49%)
Cx. quinquefasciatus	AATCT/AGATT (56.36%)	AGCAT/ATGCT (7.30%)	AACTC/AGTTG (6.27%)	AATCC/ATTGG (3.22%)
An. darlingi	AGGCC/GGCCT (8.33%)	AACGG/CCGTT (7.99%)	AAACG/CGTTT (5.56%)	ATCCG/CGGAT (4.86%)
An. albimanus	ACGCC/GGCGT (8.75%)	AGGCC/GGCCT (7.50%)	AACGG/CCGTT (6.67%)	ACCAG/CTGGT (5.42%)
An. sinensis	AACCT/AGGTT (23.81%)	AAATG/CATTT (12.70%)	AAAAC/GTTTT (7.94%)	AAAAT/ATTTT (7.94%)
An. atroparvus	AAAAT/ATTTT (10.00%)	AAATG/CATTT (7.00%)	AAACG/CGTTT (6.00%)	AAATT/AATTT (6.00%)
An. nili	AAAAC/GTTT (14.29%)	AACGG/CCGTT (14.29%)	AATAT/ATATT (14.29%)	AAAAG/CTTTT (7.14%)
An. dirus A	AAAAT/ATTTT (11.59%)	AAACG/CGTTT (9.42%)	AACCG/CGGTT (7.97%)	AAAAC/GTTTT (5.80%)
An. farauti	AATAG/CTATT (16.67%)	AAATG/CATTT (11.11%)	AAAAT/ATTTT (8.73%)	AAAAC/GTTTT (5.56%)
An. funestus	AAAAC/GTTTT (15.71%)	AAAAT/ATTTT (14.29%)	AAATT/AATTT (10.00%)	AATAG/CTATT (5.71%)
An. mininus A	AAAAC/GTTTT (12.90%)	AAAAT/ATTTT (12.90%)	AATAC/GTATT (12.90%)	AATAT/ATATT (12.90%)
An. culicifacies A	AAAAT/ATTTT (20.00%)	AAAAC/GTTTT (10.00%)	AAATG/CATTT (10.00%)	AATTC/GAATT (10.00%)
An. maculatus	AAAAC/GTTTT (17.24%)	AAACT/AGTTT (10.34%)	AAATG/CATTT (10.34%)	AAATT/AATTT (10.34%)
An. stephensi	AAAAC/GTTTT (7.14%)	AACCG/CGGTT (7.14%)	AAAAT/ATTTT (5.36%)	AAAGT/ACTTT (5.36%)
An. epiroticus	AAATT/AATTT (11.39%)	AAATG/CATTT (8.86%)	AAAAC/GTTTT (7.59%)	AAACT/AGTTT (7.59%)
An. christyi	AAAAC/GTTTT (16.28%)	AATAC/GTATT (11.63%)	AAAAT/ATTTT (4.65%)	AAAGT/ACTTT (4.65%)
An. melas	AAATG/CATTT (17.53%)	AAAAC/GTTTT (15.46%)	AATAT/ATATT (10.82%)	AAAAT/ATTTT (8.76%)
An. merus	AAATG/CATTT (20.21%)	AATAT/ATATT (13.47%)	AAAAT/ATTTT (9.84%)	AAAAC/GTTTT (8.29%)
An. quadriannulatus	AAATG/CATTT (22.54%)	AAACT/AGTTT (15.03%)	AAAAT/ATTTT (10.40%)	AATAT/ATATT (9.25%)
An. arabiensis	AAATG/CATTT (27.07%)	AATAT/ATATT (11.05%)	AAAAT/ATTTT (8.29%)	AAACT/AGTTT (7.73%)
An. gambiase	AAATG/CATTT (42.22%)	AATAT/ATATT (10.28%)	AAACT/AGTTT (7.50%)	AAAAC/GTTTT (6.67%)
An. coluzzii	AAATG/CATTT (32.30%)	AAAAT/ATTTT (10.62%)	AAAAC/GTTTT (8.41%)	AATAT/ATATT (8.41%)

	Hexanucleotide			
D. melanogaster	ACAGAT/ATCTGT (17.06%)	ACATCC/GGATGT (5.69%)	AACAGC/GCGTT (5.21%)	AATACT/AGTATT (3.32%)
Ae. albopictus	AGCATG/CATGCT (18.09%)	AATGCT/AGCATT (14.92%)	AACGCT/AGCGTT (4.14%)	AAGCAG/CTGCTT (3.32%)
Ae. aegypti	AACGCT/AGCGTT (15.25%)	AAGCTC/GAGCTT (8.58%)	AAGCAG/CTGCTT (7.26%)	AAGTCC/GGACTT (6.16%)
Cx. quinquefasciatus	AAAAAC/GTTTTT (21.79%)	AGCATG/CATGCT (12.19%)	AAAACC/GGTTTT (6.44%)	AAGCAT/ATGCTT (5.25%)
An. darlingi	AACAGC/GCTGTT (28.40%)	ACCGAG/CTCGGT (6.17%)	ACCAGC/GCTGGT (4.94%)	AAGGAG/CTCCTT (3.70%)
An. albimanus	AACAGC/GCTGTT (26.51%)	AAAGAG/CTCTTT (6.02%)	ACCAGC/GCTGGT (6.02%)	ACCGGG/CCCGGT (4.82%)
An. sinensis	AACAGC/GCTGTT (25.81%)	AAAACC/GGTTTT (6.45%)	AAGATT/AATCTT (6.45%)	AAGTAG/CTACTT (6.45%)
An. atroparvus	AACAGC/GCTGTT (31.19%)	AGCAGG/CCTGCT (7.34%)	AAAGAG/CTCTTT (4.59%)	ACCAGC/GCTGGT (4.59%)
An. nili	AAAAAT/ATTTTT (13.33%)	AACAAT/ATTGTT (13.33%)	AGAGAT/ATCTCT (13.33%)	AACGAC/GTCGTT (6.67%)
An. dirus A	AACAGC/GCTGTT (17.86%)	AAAGAG/CTCTTT (9.52%)	AAAAGG/CCTTTT (7.14%)	ACCAGC/GCTGGT (5.95%)
An. farauti	AAAAGG/CCTTTT (27.27%)	AATCGG/CCGATT (9.09%)	AAAGAG/CTCTTT (6.06%)	AACAGC/GCTGTT (6.06%)
An. funestus	AAAAGG/CCTTTT (27.03%)	AAAGAG/CTCTTT (21.62%)	AGAGAT/ATCTCT (8.11%)	AACAAG/CTTGTT (5.41%)
An. mininus A	AAGTAG/CTACTT (18.52%)	AAGTAT/ATACTT (14.81%)	AAAAAG/CTTTTT (11.11%)	AAAAGG/CCTTTT (11.11%)
An. culicifacies A	AAGGAG/CTCCTT (25.00%)	AAGAGG/CCTCTT (16.67%)	AAAAGG/CCTTTT (8.33%)	AAAATG/ATTTTC (8.33%)
An. maculatus	AAGTAG/CTACTT (23.08%)	AAAGAG/CTCTTT (19.23%)	AACAGC/GCTGTT (11.54%)	AATTAC/AATTGT (11.54%)
An. stephensi	AAGTAG/CTACTT (17.14%)	AACAAG/CTTGTT (11.43%)	AACAGC/GCTGTT (11.43%)	ACTCCG/CGGAGT (11.43%)
An. epiroticus	AACAGC/GCTGTT (27.78%)	AAAAGG/CCTTTT (8.33%)	ACCAGC/GCTGGT (6.94%)	AAAGAG/CTCTTT (5.56%)
An. christyi	AACTGT/ACAGTT (38.00%)	AACAGC/GCTGTT (18.00%)	AAAAGG/CCTTTT (8.00%)	AACAGT/ACTGTT (8.00%)
An. melas	AAAGAG/CTCTTT (24.11%)	AACAAG/CTTGTT (8.04%)	AAAAGG/CCTTTT (7.14%)	AACAGC/GCTGTT (6.25%)
An. merus	AACAGC/GCTGTT (14.47%)	AAAGAG/CTCTTT (13.84%)	AAAAGG/CCTTTT (8.81%)	AACAAG/CTTGTT (5.66%)
An. quadriannulatus	AAAGAG/CTCTTT (14.42%)	ACACAT/ATGTGT (10.58%)	AAACAT/ATGTTT (7.69%)	AAAAGG/CCTTTT (6.73%)
An. arabiensis	AACAAG/CTTGTT (9.02%)	AAAAGG/CCTTTT (7.38%)	AAAGAG/CTCTTT (7.38%)	AACAGC/GCTGTT (7.38%)
An. gambiase	AATATG/CATATT (23.14%)	AAAGAG/CTCTTT (8.73%)	AAAAGG/CCTTTT (6.55%)	AAACAT/ATGTTT (6.11%)
An. coluzzii	AATATG/CATATT (14.81%)	AAAGAG/CTCTTT (11.11%)	ACATAT/ATATGT (10.19%)	AAAAGG/CCTTTT (7.41%)
